# Integrative Transcriptomic and Phosphoproteomic Analysis Reveals Key Components of the SnRK1 Signaling Network in Rice

**DOI:** 10.1002/pld3.70120

**Published:** 2025-11-17

**Authors:** Maria C. Faria‐Bates, Chandan Maurya, K. Muhammed Jamsheer, Vibha Srivastava

**Affiliations:** ^1^ Department of Crop, Soil, and Environmental Sciences University of Arkansas System Division of Agriculture Fayetteville Arkansas USA; ^2^ Amity Institute of Genome Engineering Amity University Uttar Pradesh Noida India; ^3^ Department of Immunobiology University of Lausanne Epalinges Switzerland

**Keywords:** phosphoproteomics, protein phosphorylation, proteomics, SnRK1–TOR signaling, starvation, transcriptomics

## Abstract

SnRK1 is an evolutionarily conserved protein kinase belonging to the SNF1/AMPK family of protein kinases that is central to adjusting growth in response to the energy status. Numerous studies have shown adaptive and developmental roles of SnRK1, but the understanding of the SnRK1 signaling network in monocots is limited. Using CRISPR/Cas9 mutagenesis to target the functional kinase subunits in rice, we carried out comprehensive phenotypic, transcriptomic, proteomic, and phosphoproteomic analyses of rice *snrk1* mutants displaying growth defects under normal and starvation conditions. These analyses revealed the role of SnRK1 signaling in controlling growth and stress‐related processes in both energy‐sufficient and energy‐limited conditions and pointed to the subfunctionalization of SnRK1 kinase subunit genes. In addition to the classical protein targets of SnRK1, phosphoproteomics revealed novel targets including the key components of intracellular membrane trafficking, ethylene signaling, and ion transport. The upregulation of stress‐related processes and suppression of growth‐related processes in *snrk1* mutants correlated with their phenotypic defects. Overall, this study highlights a dual role of SnRK1 as a promoter of growth under favorable conditions and a critical regulator of adaptive response under stress conditions.

## 1 Introduction

Sucrose Non‐Fermenting Related Kinase 1 (SnRK1) plays a crucial role in coordinating growth and cellular metabolism according to energy status through phosphorylation of its target proteins in Ser/Thr sites. SnRK1 is the plant ortholog of the evolutionarily conserved SNF1/AMPK protein kinase family and is activated by starvation and other stress conditions. The hetero‐trimeric SnRK1 complex contains three subunits: the catalytic α subunit and the regulatory β and βγ subunits (Broeckx et al. 2016; Jamsheer K et al. 2021; Polge and Thomas 2007). To maintain cellular energy homeostasis and ensure the plant's survival, SnRK1 promotes catabolism and suppresses anabolism under stress (Peixoto and Baena‐González 2022; Cho et al. 2012; Pedrotti et al. 2018; Wang et al. 2021) and possibly also in the normal or energy‐sufficient conditions (Henninger et al. 2022; Wang et al. 2021). Upon activation, SnRK1 triggers signaling events that lead to the repression of anabolic processes such as cell wall formation, protein translation, and ribosome biogenesis and the induction of catabolic processes, for example, carbohydrate, lipid, and amino acid catabolism (Baena‐González et al. 2007; Henninger et al. 2022; Lu et al. 2007; Nukarinen et al. 2016; Wang et al. 2021).

It is now well established that SnRK1 controls metabolic processes through phosphorylation of key metabolic enzymes that affect their stability and/or activity. Some of the known SnRK1 targets are the sucrose‐phosphate synthases (SPS), fructose‐2,6‐bisphosphatase (F2KP), carbonic anhydrase (CA), pyruvate kinase (PK), sucrose synthase (SUS), inositol polyphosphate kinase 2 beta (IPK2β), nitrate reductase (NR), and diacylglycerol acyltransferase 1 (DGAT1) (Caldo et al. 2018; Cho et al. 2016; Kulma et al. 2004; Luo et al. 2020; Song et al. 2019; Sugden et al. 1999; Yang et al. 2018). By phosphorylating these proteins, SnRK1 regulates carbohydrate, inositol, nitrogen, and lipid metabolism. Additionally, SnRK1 regulates numerous anabolic processes through inhibition of Target of Rapamycin Complex 1 (TORC1) signaling by phosphorylating the TORC1 component, RAPTOR1 (Gwinn et al. 2008; Nukarinen et al. 2016). SnRK1 is also known to orchestrate signaling cascades by interacting with different protein kinases and phosphatases. Protein–protein interaction (PPI) networks in Arabidopsis have revealed the role of SnRK1 in defense pathways through its interaction with mitogen‐activated protein kinase 6 (MAPK6) and other MAPK domain proteins and receptor‐like kinase (RLK) family proteins (Carianopol et al. 2020; Cho et al. 2016; Jamsheer K et al. 2021). Moreover, by stimulating jasmonic acid (JA) and salicylic acid (SA) signaling in rice, SnRK1 promotes broad‐spectrum disease resistance against bacterial and fungal pathogens (Cao et al. 2024; Filipe et al. 2018). Arabidopsis SnRK1 interacts with proteins involved in biotic stress such as Phloem protein 2 A5 (PP2A5), nematode resistance proteins, HSPRO1 and HSPRO2, and directly phosphorylates nonexpressor of PR genes (NPR1) to control plant immunity (Chen et al. 2025; Jamsheer K et al. 2021). Thus, by integrating plant growth, development, and defense responses, SnRK1 acts as a signaling hub, triggering adaptive responses to promote plant survival in unfavorable conditions. Similarly, SnRK1 also regulates gene expression by phosphorylating transcription factors and chromatin‐remodeling enzymes (Chan et al. 2017; Mair et al. 2015; Wang et al. 2021).

Our knowledge of the SnRK1 signaling network in plants is mostly based on the studies conducted on Arabidopsis, and our understanding of SnRK1 signaling in monocots is rather limited. Nonetheless, research in cereals has highlighted the crucial role of SnRK1 in adaptive and developmental responses (Filipe et al. 2018; Li et al. 2020; Wang et al. 2021; Yang, Huang, et al. 2024). In rice, Lu et al. (2007) demonstrated that OsSnRK1αA regulates germination and seedling growth by phosphorylating the MYBS1 transcription factor, which activates genes involved in starch degradation and glucose metabolism. This regulation ensures efficient energy production and carbohydrate mobilization to support early seedling development. Several studies showed that disruption of SnRK1 signaling leads to lower seed set, abnormal seed development, seed abortion, and lower seed filling in cereal crops (Bledsoe et al. 2017; Cao et al. 2024; Hu et al. 2022; Zhang et al. 2001). In particular, Hu et al. (2022) showed that SnRK1 is essential for rice grain filling by promoting nonstructural carbohydrate transport from sheaths to panicles and that loss of *OsSnRK1αA* disrupts this process, leading to starch accumulation in sheaths and reduced yield. In wheat, the SnRK1α (TaSnRK1α) regulates starch biosynthesis by modulating AGPase activity (Kumar et al. 2024), whereas in maize, SnRK1 interacts with trehalose‐6‐phosphate to influence early kernel development and prevent stress‐induced kernel abortion (Bledsoe et al. 2017). Collectively, these findings demonstrate that SnRK1 is a conserved regulator of sugar metabolism, reproductive development, and yield determination in cereals. However, to map the comprehensive network of SnRK1 signaling and resolve its conservation and diversity across the plant lineage, studies focusing on SnRK1 signaling networks in monocots are needed. This information is also important in the deeper understanding of the SnRK1‐dependent signaling network and its relationship with growth and adaptive responses in plants.

In this study, we focused on the three functional kinase subunits of SnRK1 to systematically analyze the SnRK1‐dependent signaling network in rice. We investigated the role of the SnRK1 signaling in the growth and adaptation of rice seedlings to starvation through phenotypic characterization of rice SnRK1 mutants developed through CRISPR/Cas9 mutagenesis and their transcriptomic, proteomic, and phosphoproteomic analyses. We found that SnRK1 plays a major role in normal growth and development, energy starvation, and defense against rice blast fungus (*Magnaporthe oryzae*). Through transcriptomic analysis, we identified the SnRK1‐dependent gene networks involved in the developmental processes and adaptive responses to starvation. Through proteomics and phosphoproteomics, we identified protein networks regulated by SnRK1 under starvation. Further, along with the classical conserved targets such as RAPTOR, we identified potentially novel phosphosites of the SnRK1 signaling network in rice. Together, these findings highlight the central role of SnRK1 as a hub for coordinating not only stress and defense responses but also energy‐driven growth processes in rice and possibly other plant species.

## 2 Results

### 2.1 SnRK1 Is a Key Regulator of Plant Growth and Defense in Rice

In a previous study, we developed rice mutants of SnRK1 kinase subunits through CRISPR/Cas9 mutagenesis (Pathak et al. 2022). Two sets of *snrk1* mutants were developed based on sequence homology of the three functional paralogs of the SnRK1 kinase α‐subunit genes (*OsSnRK1α*): *OsSnRK1αA* (Os05g0530500/LOC_Os05g45420), *OsSnRK1αB* (Os03g0289100/LOC_Os03g17980), and *OsSnRK1αC* (Os8g0484600/LOC_Os08g37800). Of these, *OsSnRK1αB* and *OsSnRK1αC* show high sequence homology (88.2% genomic and 98.02% protein sequence), whereas *OsSnRK1αA* is more divergent. Based on this, single mutants of *OsSnRK1αA* and double mutants of *OsSnRK1αBC* were developed using the *japonica* rice variety, Kitaake, and the characterized homozygous lines of *ossnrk1αa* and *ossnrk1αbc* were selected for this study. These mutants are referred to as *snrk1a* and *snrk1bc*, hereafter. Both mutants contain a truncation in the kinase domain through incorporation of an early stop codon (File S2). Gene expression analysis by qPCR verified that the nontargeted paralog(s) of *OsSnRK1α* were not affected whereas the targeted paralog(s) were downregulated in each mutant (Figure S1), indicating degradation of the nonsense transcript, a conserved gene regulatory mechanism in eukaryotes, and confirming the absence of collateral effects on the nontargeted paralogs.

**FIGURE 1:**
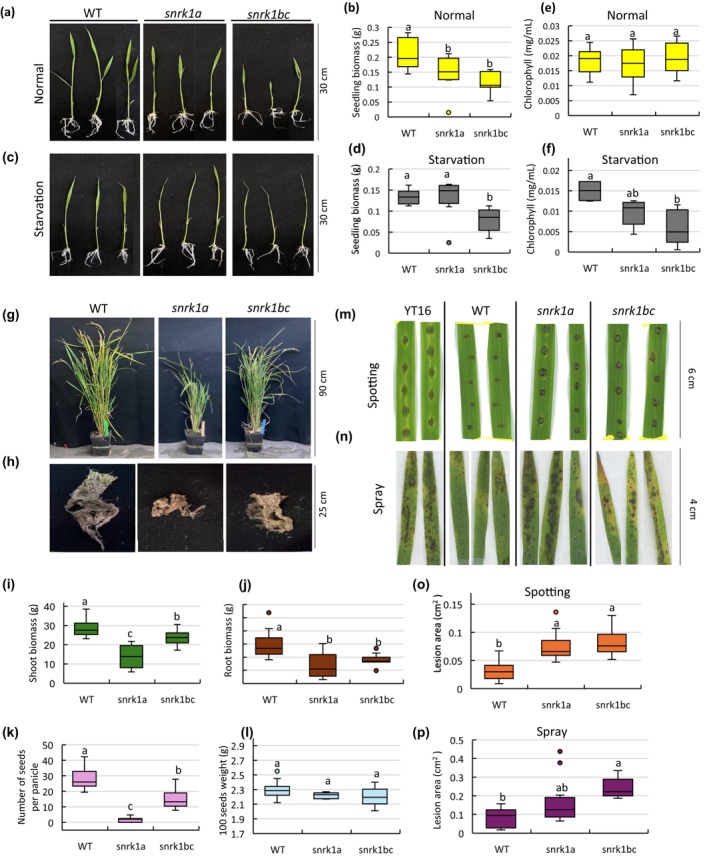
Phenotypic characterization of *snrk1* mutants. (a–d) Seedling phenotype and biomass of 9‐day‐old seedlings of wildtype Kitaake (WT), *snrk1a*, and *snrk1bc* grown on half‐strength MS medium under a 14‐h photoperiod (normal condition) (a, b) or exposed to 48 h of extended darkness after 7 days of normal growth (starvation) (c, d). (e, f) Chlorophyll content in 9‐day‐old seedlings of WT, *snrk1a*, and *snrk1bc* grown under normal conditions (e) or subjected to starvation (f). (g–l) Representative plants of WT, *snrk1a*, and *snrk1bc* at maturity in the greenhouse. Aboveground shoot (g), roots (h), shoot and root biomass (i, j), reproductive or yield traits, number of seeds per panicle (k), and weight of 100 seeds (l). (m–p) Disease response of detached leaves following inoculation with *M. oryzae* strain Guy11. Two types of assays using spotting (m) or spray (n) of the inoculum are shown in which YT16 serves as the susceptible check. Lesion area in the mutants in spotting (o) and spray (p) assays is compared to WT Kitaake. Significant differences (*p* < 0.05) by one‐way ANOVA followed by Tukey's HSD test are shown by small letters. Error bars represent standard deviation (SD). *n* = 10 (a–f), 20 (g–l), 18 (o), and 27 (p).

To understand the role of SnRK1 in seedling growth, the *snrk1a* and *snrk1bc* mutants were cultivated along with the wildtype (WT). Defective growth was observed in *snrk1*seedlings grown on half‐strength MS media under a 14 h photoperiod, representing normal conditions (energy sufficiency) as well as in seedlings exposed to 48 h of extended darkness inducing starvation (energy deficiency). In normal conditions, 9‐day‐old seedlings of *snrk1a* and *snrk1bc* mutants developed shorter length and lower biomass (shoot and root) compared to the wildtype (WT) (Figures 1a,b and S2a–d). Under starvation, on the other hand, both *snrk1a* and *snrk1bc* seedlings appeared thinner than WT (Figure 1c). Shoot and root lengths were not significantly different (Figure S2e,f); however, reduced biomass was observed in *snrk1bc* seedlings (Figures 1d and S2g,h), corroborating with the development of very thin seedlings consisting of reduced leaf width and tissue thickness as well as yellowing in the culm and leaf rolling (Figure S3). Next, chlorophyll content of the seedlings under normal conditions was not significantly different between the three genotypes (Figure 1e), but under starvation, both *snrk1* mutants accumulated lower chlorophyll with *snrk1bc* seedlings showing a significant reduction (Figure 1f). These observations indicate the role of SnRK1 signaling in rice seedling establishment and their response to starvation, as well as potential subfunctionalization of *OsSnRK1α* genes. The mature plants of *snrk1a* and *snrk1bc* mutants raised in greenhouse conditions also showed growth defects (Figure 1g,h). Both *snrk1* mutants showed lower shoot and root biomass (Figure 1i,j). Reduced fertility in *snrk1* mutants was indicated by a lower number of seeds per panicle, whereas the weight of 100 grains was not significantly different (Figure 1k,l). Here, *snrk1a* plants showed more severe reductions in biomass and fertility, indicating important roles for SnRK1 signaling in the vegetative and reproductive development of the plant. These results further confirm the functional diversification of *OsSnRK1α* genes.

SnRK1 plays an important role in defense against pathogens (Filipe et al. 2018). Therefore, we analyzed the disease response in *snrk1* mutants by spotting or spraying detached leaves with rice blast fungus, *Magnaporthe oryzae* strain Guy11, that causes diamond‐shaped lesions with a gray center (sporulating lesions) leading to leaf blast disease in susceptible cultivars such as YT16 that show high susceptibility to *Magnaporthe oryzae*, as it carries the susceptible pi‐ta allele (Bryan et al. 2000). In both assays, snrk1 mutants showed enhanced susceptibility compared to WT Kitaake (Figure 1m–p). As expected, YT16 showed diamond‐shaped, coalescing lesions with surrounding chlorosis in the spotted area. The WT (cv. Kitaake), on the other hand, showed small, round lesions confined to the spotted area, a characteristic of hypersensitive response consisting of localized cell death. The *snrk1* mutants, however, showed larger, diamond‐shaped lesions, consisting of clear gray centers and a significant increase in the lesion size (Figure 1m,o). Similar results were obtained in the spray assay with significant differences in lesion areas between WT and *snrk1* mutants (Figure 1n,p).

In summary, growth defects in *snrk1* mutants in the energy‐sufficient and energy‐deficient states and their enhanced susceptibility to leaf blast disease indicate regulatory roles of SnRK1 signaling in normal growth processes, metabolic stress, and disease response in rice. To validate the *snrk1* mutation with the plant phenotype, two additional mutant lines representing the second allele of *ossnrk1αa* and *ossnrk1αbc* each were analyzed (File S3). These mutants referred to as *snrk1a.2* and *snrk1bc.2* showed similar phenotypic defects in the seedlings cultivated in normal or starvation conditions on MS media and mature plants grown in the greenhouse as well as enhanced susceptibility to *M. oryzae* (Figure S4).

### 2.2 SnRK1 Plays a Regulatory Role in Energy‐Sufficient State

**FIGURE 2:**
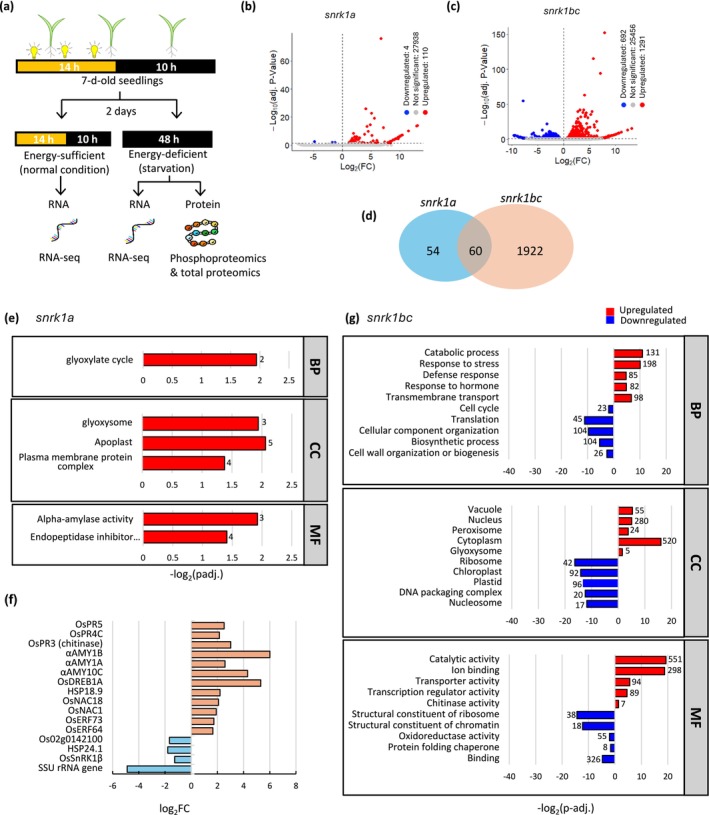
Experimental set up and transcriptomic response of *snrk1* mutants under energy‐sufficient (normal) conditions. (a) Experimental setup for transcriptomic, proteomic, and phosphoproteomic analyses of *snrk1* mutants. Seven‐day‐old seedlings grown in 14‐h photoperiod on half‐strength MS media were divided into two treatment groups: (i) Energy‐sufficient (normal) condition, where seedlings were maintained under 14‐h photoperiod for two additional days, and (ii) energy‐deficient (starvation) condition, where seedlings were transferred to complete darkness for 2 days (48 h) starting 12 noon. Total RNA was extracted from seedlings in normal and starvation conditions for transcriptomic analysis, and total protein was extracted from seedlings subjected to starvation for proteomic and phosphoproteomic analysis. (b, c) Volcano plots showing differentially expressed genes (DEGs) in *snrk1a* (b) and *snrk1bc* (c) compared to WT in energy‐sufficient condition. Red dots indicate upregulated genes, and blue dots indicate downregulated genes (*p* adj. ≤ 0.05, |log_2_FC| > 0). (d) Venn diagram showing unique and overlapping DEGs between *snrk1a* and *snrk1bc* in 9‐day‐old seedlings under energy‐sufficient state. (e) Enrichment of Gene Ontology (GO) terms in upregulated DEGs in *snrk1a* mutant. Enriched biological process (BP), cellular component (CC), and molecular function (MF) are shown. (f) Log_2_ fold change of selected DEGs (*p* adj. ≤ 0.05, |log_2_FC| > 1) in *snrk1a* based on RNA‐seq analysis, including upregulated stress‐related transcription factors (e.g., *OsERF64*, *OsNAC1*, and *OsDREB1A*), pathogenesis‐related genes (*OsPR4C* and *OsPR3*), α‐amylase genes (*αAMY10C*, *αAMY1A*, and *αAMY1B*), and the downregulated genes, including the β‐subunit of *OsSnRK1*, *SSU rRNA*, *HSP24.1*, and *Os02g0142100*. (g) GO enrichment analysis of DEGs in *snrk1bc* mutant. Red bars represent upregulated GO terms, and blue bars represent downregulated GO terms (*p* adj. ≤ 0.05).

As described above, *snrk1* mutants displayed growth defects under normal and starvation conditions, suggesting a broad role of SnRK1 signaling in rice seedling growth. To better understand the underlying processes, we conducted RNA‐seq analysis on WT, *snrk1a*, and *snrk1bc* seedlings under normal and starvation conditions (Figure 2a). Principal component analysis revealed a greater variation of gene expression between *snrk1bc* and WT than between *snrk1a* and WT under normal conditions (Figure S5a,b). Accordingly, in comparison to WT, 114 and 1982 differentially expressed genes (*p* adj. < 0.05) were observed in *snrk1a* and *snrk1bc*, respectively (Figure 2b–c and Table S1 and S2), 60 of which were common to the two mutants (Figure 2d). Of these 60 genes, 56 are similarly regulated: 55 are upregulated, and 1 is downregulated in both mutants, indicating overlapping roles of OsSnRK1A, OsSnRK1B, and OsSnRK1C in SnRK1 signaling.

The gene ontology (GO) enrichment analysis in *snrk1a* showed the association of upregulated genes with the glyoxylate cycle in biological process (BP), glyoxysome, apoplast and plasma membrane protein complex terms in cellular component (CC) and α‐amylase and endopeptidase inhibitor activities in molecular function (MF) (Figure 2e and Table S3). However, other well‐known stress‐responsive genes such as *OsERF64*, *OsERF73*, *OsNAC1*, *OsNAC18*, *HSP18.9*, and *OsDREB1A* were also upregulated (*p* adj. ≤ 0.05, log_2_FC > 1), along with three pathogenesis‐related genes, *OsPR3*, *OsPR4C*, and *OsPR3* (chitinase) and 3 *αAMY* genes (*αAMY10C*, *αAMY1A*, and *αAMY1B*). Only four genes were significantly downregulated (*p* adj. ≤ 0.05, log_2_FC < 1) in the *snrk1a* mutant that included the β‐subunit of *OsSnRK1*, small subunit (SSU) *rRNA* gene, *HSP24.1*, and *Os02g0142100* (Figure 2f). In *snrk1bc*, on the other hand, several BP terms were associated with upregulated or downregulated genes (Table S4). Notably, stress‐related BPs were upregulated, whereas growth‐related BPs were downregulated. The upregulated processes included catabolic process, response to stress, and defense response, and downregulated processes included cell cycle, translation, and biosynthetic process. Accordingly, CC terms associated with upregulated genes included vacuole and peroxisome, and those with downregulated genes included ribosome, plastids, and nucleosome. Similarly, MF terms, catalytic activity, ion binding, and chitinase activity were associated with upregulated genes and structural constituents of ribosome and chromatin were associated with downregulated genes (Figure 2g). Taken together, these results indicate that under normal conditions, SnRK1 is involved in promoting growth‐related processes and suppressing stress‐related processes. Collectively, the phenotypic and transcriptomic analyses indicate an important role of SnRK1 in normal growth and development. A higher number of deregulated processes in *snrk1bc*, especially the downregulation of growth processes, align with the phenotype of a greater reduction of shoot and root biomass in the *snrk1bc* mutant compared to the *snrk1a* mutant (Figure 1a,b).

### 2.3 Starvation‐Response in *snrk1* Mutants Is Deregulated

**FIGURE 3:**
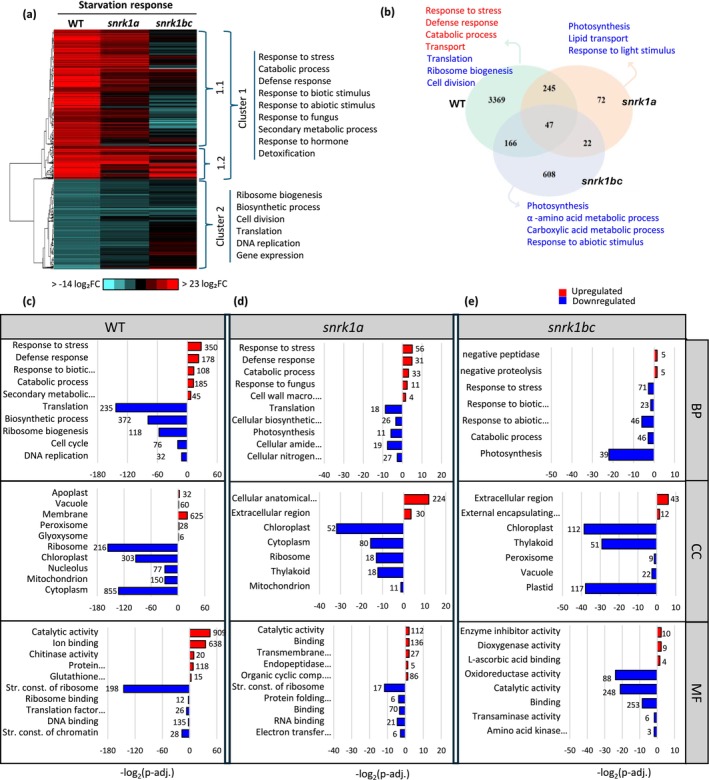
Transcriptomic changes in *snrk1* mutants under starvation. (a) Hierarchical clustering of 3827 differentially expressed genes (DEGs) (*p* adj. < 0.05, |log_2_FC| > 0) in 9‐day‐old seedlings of WT, *snrk1a*, and *snrk1bc* in starvation. The upregulated and downregulated gene clusters in the WT are shown as Clusters 1 and 2, respectively. Cluster 1 is divided into two subclusters, 1.1 and 1.2, to indicate deregulated genes (1.1) or unaltered genes (1.2) within cluster 1 in *snrk1bc* mutant. Major GO terms associated with the clusters are indicated. (b) Venn diagram showing the number of unique and common DEGs among WT, *snrk1a*, and *snrk1bc* during starvation. Gene ontology (GO) processes associated with the unique genes in each genotype are indicated with upregulated GO terms shown in red and downregulated in blue. (c–e) Functional enrichment analysis of DEGs showing enriched gene ontology (GO) terms in biological process (BP), cellular component (CC), and molecular function (MF) in WT (c), *snrk1a* mutant (d), and *snrk1bc* mutant (e). Red and blue bars indicate upregulated and downregulated terms, respectively, with numbers of genes associated with each term indicated on each bar. All terms shown are significant at *p* adj. ≤ 0.05.

SNF1/SnRK1 signaling plays a central role in adaptation to stress and starvation in yeast and Arabidopsis (Baena‐González and Sheen 2008; Coccetti et al. 2018; Hedbacker and Carlson 2008; Polge and Thomas 2007). To identify starvation‐triggered processes in our *snrk1* mutants, we compared the transcriptome of 9‐day‐old seedlings under extended darkness, representing a starvation/energy‐deficient state with that in the normal/energy‐sufficient state (Figure 2a). Principal component analysis showed significant differences between the transcriptomes of *snrk1a*, *snrk1bc*, and WT under starvation (Figure S5c,d). A total of 3827 genes were differentially expressed (*p* adj. ≤ 0.05) in WT during starvation, whereas *snrk1a* and *snrk1bc* mutants showed only 386 and 843 differentially expressed genes, respectively (Figure S6 and Table S5–S7). Hierarchical clustering of these genes showed upregulated (2397 genes) and downregulated (1430 genes) clusters in WT, representing starvation‐induced and repressed processes. Starvation‐induced genes (Clusters 1.1 and 1.2) are associated with stress and defense responses and starvation‐repressed genes (Cluster 2) with energy‐driven growth processes (Figure 3a). In WT, 1048 genes were upregulated greater than or equal to fourfold, whereas only 36 were downregulated greater than or equal to fourfold, suggesting that the starvation response involves marked upregulation of a large set of genes (Table S5). In *snrk1* mutants, several genes in each cluster were deregulated as the number of unique genes in each genotype varied, and only 47 genes were common, indicating specific roles of *OsSnRK1* kinase subunit genes and a unique set of upregulated and downregulated processes in each genotype (Figure 3b).

In the WT, stress and catabolic processes were upregulated, and corroborating with that, CC terms, vacuole, peroxisome, glyoxysome and MF terms, catalytic activity, ion binding, and chitinase activity were associated with upregulated genes during starvation. On the other hand, growth‐related processes and CC terms, ribosome and chloroplast, and MF terms, structural components of ribosome were associated with downregulated genes during starvation (Figure 3c and Table S8). The *snrk1a* mutant showed a similar pattern of gene enrichment, but a far lower number of genes were associated with each process (Figure 3d and Table S9). However, the *snrk1bc* mutant showed an inverse pattern of starvation‐triggered processes. First, Cluster 1 consisted of two subclusters, 1.1 and 1.2 (Figure 3a). Although the enrichment of GO processes was similar in the two subclusters, 1.1 generally consisted of deregulated genes (SnRK1‐dependent), whereas 1.2 consisted of unaltered genes (SnRK1‐independent) in the *snrk1bc* mutant. Next, response to stress and catabolic processes was downregulated in *snrk1bc* seedlings, and negative regulation of peptidase/proteolysis was upregulated. Accordingly, genes associated with CC terms, chloroplast and peroxisome, and MF terms, oxidoreductase activity, and catalytic activity were downregulated (Figure 3e and Table S10). These findings corroborate with Arabidopsis studies that showed *snrk1* mutants are unable to turn on stress signaling or respond to starvation (Baena‐González et al. 2007; Henninger et al. 2022; Pedrotti et al. 2018). These findings also provide mechanistic cues for growth defects in *snrk1* mutants that display abnormally induced starvation‐triggered processes in an energy‐sufficient state, potentially creating a paucity of energy supply, compromising the energy‐driven growth processes. It should be noted that *SnRK1* genes are not transcriptionally induced during extended darkness. However, the two rice homologs of *DARK INDUCIBLE 6/ASPARAGINE SYNTHASE 1* (*DIN6/ASN1*), a major marker of Arabidopsis SnRK1 signaling are differentially regulated, and *OsASN1* is induced, whereas *OsASN2* is repressed during starvation (Figure S7).

### 2.4 Differential Phosphorylation of Peptides in *snrk1bc* Mutant

**FIGURE 4:**
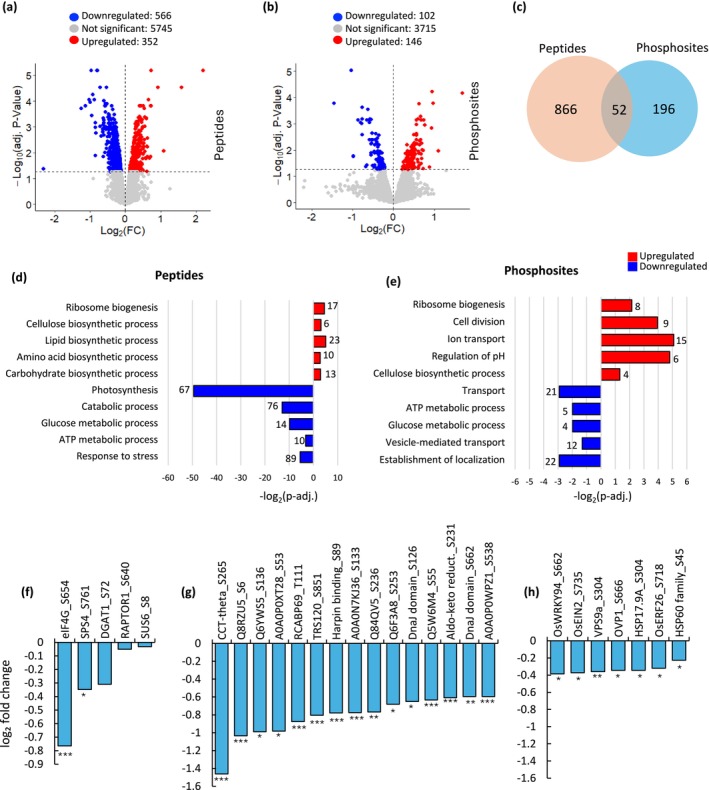
Proteomic and phosphoproteomic analyses of *snrk1bc* mutant under starvation. (a, b) Volcano plots of differentially abundant peptides (a) and phophosites (b) in *snrk1bc* compared to WT. Each dot represents a peptide/phosphosite, with the *x* axis indicating log_2_ fold change and the *y* axis showing significance. Differentially abundant peptides/phosphosites (*p* adj. < 0.05 and |log_2_ fold change| > 0) are shown in red for upregulated, blue for downregulated, and gray for nonsignificant change. (c) Venn diagram showing common or unique proteins detected in proteomic and phosphoporteomic analyses. (d, e) Enriched gene ontology (GO) biological processes associated with upregulated (red) or downregulated (blue) peptides (d) and phosphosites (e) in the *snrk1bc* (*p* adj. < 0.05). (f–h) Changes in the phosphorylation levels of the known SnRK1 targets (f), novel targets that show at least 1.5‐fold change (g), and selected targets identified among downregulated phosphosites in *snrk1bc* (h). Significance of downregulation for each phosphosite is indicated (*p adj. ≤ 0.05, **p adj. ≤ 0.01, ***p adj. ≤ 0.001).

To identify phosphosites regulated by SnRK1 signaling, phosphoproteomic analysis was carried out on seedlings under starvation. Additionally, total proteomic analysis was carried out to identify the effect of SnRK1 signaling at the proteome level and to correlate protein phosphorylation with protein abundance. We selected the *snrk1bc* mutant for this analysis based on the phenotypic and transcriptomic data that showed severe growth defects in its seedlings and a greater number of deregulated genes in comparison to WT. As described above, 9‐day‐old seedlings exposed to 48 h of continuous darkness were subjected to proteomic and phosphoproteomic analysis (Figure 2a). A comparison of *snrk1bc* versus WT showed 918 differentially abundant peptides and 248 differentially abundant phosphosites representing 220 proteins (Figure 4a–b and Tables S11 and S12). Fifty two of these proteins were common in the two datasets (Figure 4c), which could be used to understand the effect of phosphorylation on protein abundance. The enrichment analysis of 352 upregulated and 566 downregulated peptides showed upregulation of growth‐related processes such as ribosome biogenesis and biosynthetic processes related to cellulose, lipid, amino acids, carbohydrate, and downregulation of energy generation processes such as glucose and ATP metabolic processes, catabolic processes, and response to stress (Figure 4d). Among the 248 differentially abundant phosphosites, 102 showed reduced phosphorylation (downregulated), and the remaining 146 showed enhanced phosphorylation (upregulated) in the *snrk1bc* mutant (Figure 4b and Table S12). These phosphosites are associated with a functional network consisting of metabolic, growth, and stress processes (Figure S8 and Table S13). Notably, upregulated phosphosites are associated with growth processes such as cell division, ribosome biogenesis, and cellulose biosynthesis and the downregulated phosphosites with energy generation such as glucose metabolic processes, ATP generation, and membrane transport (Figure 4e and Table S13). In summary, both proteomic and phosphoproteomic analyses show upregulation of growth‐related processes and downregulation of catabolic and stress‐related processes in the *snrk1bc* mutant during starvation.

Of the known SnRK1 targets, Sucrose Phosphate Synthase 4 (SPS4) (Sugden et al. 1999), eukaryotic translation initiation factor 4G (eIF4G) (Cho et al. 2019), Raptor 1 (Gwinn et al. 2008; Nukarinen et al. 2016), Sucrose Synthase 6 (SUS6) (Luo et al. 2020), and Diacylglycerol O‐acyltransferase 1 (DGAT1) (Caldo et al. 2018) were downregulated (Figure 4f). These observations indicate deregulation of SnRK1 signaling in the *snrk1bc* mutant. Further, 17 phosphosites were reduced ≤ 1.5‐fold, which included eIF4G and putative novel targets: Trafficking Protein Particle Complex II‐specific subunit 120 homolog (TRS120), CCT‐theta, Harpin binding protein 1, Chlorophyll a‐b binding protein (RCABP69), DnaJ domain containing protein, Aldo‐keto reductase 2, and LOC_Os11g43950 protein (Q2R031) (Figure 4g). These phosphosites could be the direct or indirect (through another intermediary protein) targets of phosphorylation by SnRK1. In the protein abundance data (Table S11), RCABP69, Aldo‐keto reductase 2, and Harpin binding protein 1 were reduced in the *snrk1bc* mutant (*p* adj. ≤ 0.05), indicating they are stabilized by phosphorylation. Finally, downregulation of the following phosphosites is also noteworthy: transcription factor, OsWRKY94, two major regulators of ethylene signaling, ETHYLENE INSENSITIVE 2 (OsEIN2) and ETHYLENE RESPONSE FACTOR 46 (OsERF46), a major regulator of membrane transport, VPS9a, and two heat shock proteins, HSP17.9A and HSP60 family protein, and vacuolar H^+^ translocating pyrophosphatase (OVP1) (Figure 4h).

### 2.5 Glucose Metabolic Process and Energy Generation Is Regulated by SnRK1 During Starvation

**FIGURE 5:**
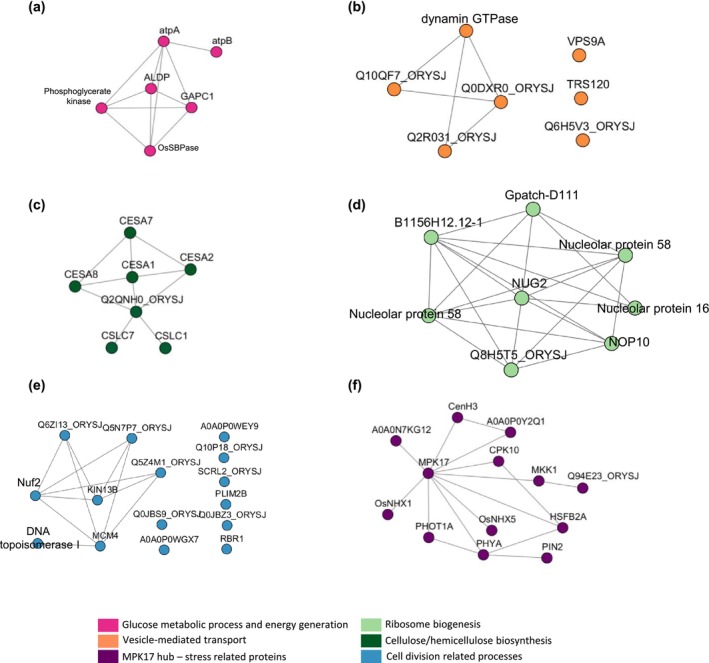
Network of differentially abundant phosphosites in the *snrk1bc* mutant compared to WT under starvation. Each cluster is associated with gene ontology (GO) biological processes color coded at the bottom. (a, b) Downregulated phosphosites involved in glucose metabolic processes and energy generation (a) or in vesicle‐mediated transport (b). **(c‐e)** Upregulated phosphosites involved in growth processes: cellulose biosynthesis (c), ribosome biogenesis (d), and cell division‐related processes (e). (f) Upregulated phosphosites associated with a stress‐related protein network with MPK17 as the hub.

The network of differentially abundant phosphosites in the *snrk1bc* mutant showed distinct clusters of downregulated and upregulated processes including the glucose metabolic process and energy generation (Figure 5a). Starch biosynthesis and degradation are regulated by photoperiod. During nighttime, starch is degraded to glucose, which is utilized through glycolysis, the citric acid cycle, and oxidative phosphorylation to produce energy (ATP). The phosphosites of key enzymes in glycolysis/gluconeogenesis and the Calvin cycle within the cluster were differentially abundant in the *snrk1bc* mutant. Specifically, Fructose‐bisphosphate aldolase 1 (ALDP), chloroplastic; Sedoheptulose 1,7‐bisphosphatase (OsSBPase); Glyceraldehyde‐3‐phosphate dehydrogenase 1 (GAPC1), cytosolic; and phosphoglycerate kinase were significantly downregulated (*p* adj. < 0.001). Similarly, the key enzymes in ATP synthesis, ATP synthase subunit alpha, chloroplastic (atpA), and ATP synthase subunit beta, chloroplastic (atpB), were also significantly downregulated (*p* adj. < 0.0001). The protein abundance of these phosphosites was reduced (*p* adj. ≤ 0.05), indicating SnRK1‐mediated phosphorylation leads to their stabilization. Together these analyses indicate that ATP generation through the glycolytic process during starvation is regulated by SnRK1 signaling in rice seedlings.

### 2.6 A Potential Role of SnRK1 Signaling in Membrane Trafficking

Membrane trafficking is necessary for the integrity of the cell at all points of growth regardless of normal or stress conditions. It involves export and import of material and the movement of cargo to cellular compartments through complex endomembrane networks including vesicles. During energy deficiency, membrane trafficking is altered to conserve and generate energy from cellular components, inducing autophagy (Feyder et al. 2015; Gill et al. 2021; Zeng et al. 2023). Phosphosites associated with vesicle‐mediated transport were affected in *snrk1bc* mutant (*p* adj. < 0.05). Of the seven downregulated phosphosites in this process (Figure 5b), two are well‐known components of vesicle generation and tethering to the target compartment: vacuolar protein sorting‐associated protein 9A (VPS9a) and trafficking protein particle complex II (TRAPPII)‐specific subunit 120 (TRS120) homolog.

VPS9a is a major GDP‐GTP exchange factor (GEF) and functional activator for Rab5a small GTPase that regulates initial steps of endocytosis (Carney et al. 2006; Nielsen et al. 2008). For example, in rice endosperm, the transport of dense vesicles from Golgi to protein storage vacuoles (PSV) is regulated by Rab5a homolog Glutelin Precursor 4 (GLUP4) and VPS9a homolog GLUP6. The *glup4* and *glup6* mutants of rice accumulate high amounts of storage proteins in the space between invaginating plasma membrane and the cell wall (Fukuda et al. 2011, 2013). Downregulation of VPS9A/GLUP6 in the phosphoproteome of *snrk1bc* mutant indicates a role for SnRK1 signaling in Rab5a/GLUP4‐mediated vesicular transport in rice seedlings. In support of this, Yu et al. (2025) recently showed that Arabidopsis VPS9a plays a key role in nutrient stress adaptation, a core function of SnRK1 signaling, by coordinating endosomal trafficking, autophagy, and nitrogen metabolism, particularly by modulating the glutamine synthetase/glutamate synthase (GS/GOGAT) cycle under starvation. Previously, van Leene et al. (2022) proposed Arabidopsis homolog of VPS9a as the true target of SnRK1. Regulation of VPS9a through phosphorylation is also substantiated by the studies that showed other GEFs such as Rho family and Rab35 GEFs are phosphorylated at tyrosine and/or serine residues (Kulasekaran et al. 2015; Patel and Karginov 2014). In our dataset, VPS9a/GLUP6 phosphorylation of serine residue was affected (*p* adj. = 0.006).

Similarly, phosphorylation of the serine residue (S851) in TRS120, a TRAPPII‐specific subunit, was downregulated in the *snrk1bc* mutant during starvation (*p* adj. < 0.001). TRAPPII is a highly conserved regulator of membrane traffic in both endocytosis and exocytosis pathways (Ravikumar et al. 2017). In Arabidopsis, it is essential for cytokinesis or cell plate formation during cell division. Accordingly, Arabidopsis TRS120 null mutants are seedling lethal due to severe cytokinesis defects (Thellmann et al. 2010). Just as in yeast, the Arabidopsis TRAPPII complex functions as a GEF for the RabA2 clade of plant Rab GTPases (Kalde et al. 2019). In Arabidopsis, TRS120 is subject to phosphorylation by BIN2, an Arabidopsis SHAGGY‐like kinase (Wiese et al. 2024). BIN2 is inhibited by the TOR pathway involving phosphorylation by Ribosomal Protein S6 Kinase (S6K) (Xiong et al. 2017). Thus, the downregulation of the TRS120 phosphosite could be explained by the upregulated TOR signaling in the *snrk1bc* mutant leading to the inactivation of SHAGGY‐like kinase(s).

### 2.7 SnRK1 Suppresses TOR Signaling and Energy‐Driven Growth Processes

The upregulated phosphosites in *snrk1bc* mutant represent indirect targets of SnRK1. These were predominantly associated with growth processes such as ribosome biogenesis, cellulose biosynthesis, and cell division (Figure 5c–e) and possibly related to upregulated TOR signaling in the mutant. Ribosome biogenesis and translation are among the conserved TOR‐dependent processes controlled by phosphorylation of S6K that in turn phosphorylates ribosomal protein S6 (RPS6) and eukaryotic translation initiation factor eIF3 and eIF5A (Magnuson et al. 2012; Mahfouz et al. 2006; Schepetilnikov et al. 2013). Earlier Nukarinen et al. (2016) reported enhanced phosphorylation of RPS6 and eIF5A in Arabidopsis *snrk1* mutants. By suppressing TOR signaling through phosphorylation of Raptor 1, SnRK1 antagonistically regulates RPS6, eIF3 subunits, and eIF5A. In *snrk1bc*, phosphosites in RPS6 (Q75LR5 and Q6Z3C2) as well as in eIF5A2 (Q2QQ48) and eIF5B (Q0DFG2) were upregulated (*p* < 0.05). eIF3B (Q8S7Q0), eIF3G (Q6K4P1), eIF3M (Q0JFH5), eIF4A‐1 (P35683), and eIF6 (Q8GVF5) were also upregulated, although not significantly (Figure S9). These observations serve as molecular evidence for the induced TOR activity in the *snrk1bc* mutant.

Among the highly significant upregulated phosphosites (*p* adj. < 0.02; log_2_FC ≥ 0.585), plasma membrane ATPase, auxin efflux carrier component 2 (OsPIN2), and ammonium transporters (OsAMT1) are noteworthy as they indicate that metabolic activities, nutrient uptake, and root development are antagonistically regulated by SnRK1 signaling. The plasma membrane ATPase regulates H^+^ gradient and assists in nutrient uptake (Falhof et al. 2016). In fact, three plasma membrane ATPases (Q7XPY2, Q8L6I2, and Q8L6I1) and the two OsAMT1s (Q6K9G1 and Q7XQ12) that play a major role in ammonium uptake were significantly upregulated in *snrk1bc* mutant (Table S12). Next, our data show that cellulose biosynthesis is antagonistically regulated by SnRK1 signaling and the upregulation of cell division and cellulose biosynthetic process is another evidence of the unchecked TOR activity in *snrk1bc* mutant during starvation. The TOR pathway is integral to cell division, and it likely promotes biosynthesis and deposition of cell wall components such as cellulose and hemicelluloses (Calderan‐Rodrigues and Caldana 2024). The phosphosites in four cellulose synthases (CESA1, CESA2, CESA7, and CESA8) and two hemicellulose biosynthesis enzymes (CSLC1 and CSLC7) were upregulated in *snrk1bc* mutant (Table S12), suggesting an indirect role of SnRK1 in controlling the biosynthesis of the cell wall components (Figure 5c). In addition, growth‐related processes such as ribosome biogenesis and cell division were upregulated in the phosphoproteome of the *snrk1bc* mutant (Figure 5d,e), which is known to activate protein synthesis and cell proliferation under nutrient‐rich conditions. Next, a cluster of phosphosites associated with stress‐related proteins was differentially regulated that consisted of mitogen‐activated protein kinase17 (OsMPK17) as the hub protein in the module (Figure 5f). OsMPK17 is a negative regulator of defense response against *Xanthomonas oryzae* pv. *Oryzae* (*Xoo*), and it interacts with MAP kinase kinase1 (MKK1), the positive regulator of defense response against *Xoo* (Yang, Zhu, et al. 2024; Zhu et al. 2022). Although the direct interaction of MPK17 and MKK1 has not been shown, the MAP kinase cascade has been implicated in the resistance response against pathogens including *Xoo* (Park et al. 2010). This module also contains light receptors, phytochrome A (PhyA) and phototropin 1 (Phot1A), that are phospho‐upregulated. PhyA and phot1A sense far‐red and blue light, respectively, and promote photomorphogenesis. Autophosphorylation of PhyA in Ser residues is a key mechanism for attenuating PhyA signaling. In rice, Ser (S599) in the hinge region of PhyA is subject to phosphorylation (Zhou et al. 2018). Phosphorylation of the same Ser (S599) is upregulated in *snrk1bc* mutant (Table S12), suggesting an indirect role of SnRK1 in regulating PhyA apoprotein. Overall, phosphoproteomics aligns with transcriptomic analysis of *snrk1bc* as both show upregulation of growth processes in the *snrk1bc* mutant during starvation and point to the role of SnRK1 signaling in conserving energy by suppressing growth and inducing catabolic processes during stress.

### 2.8 SnRK1 Phosphorylation Motifs in Rice

**FIGURE 6:**
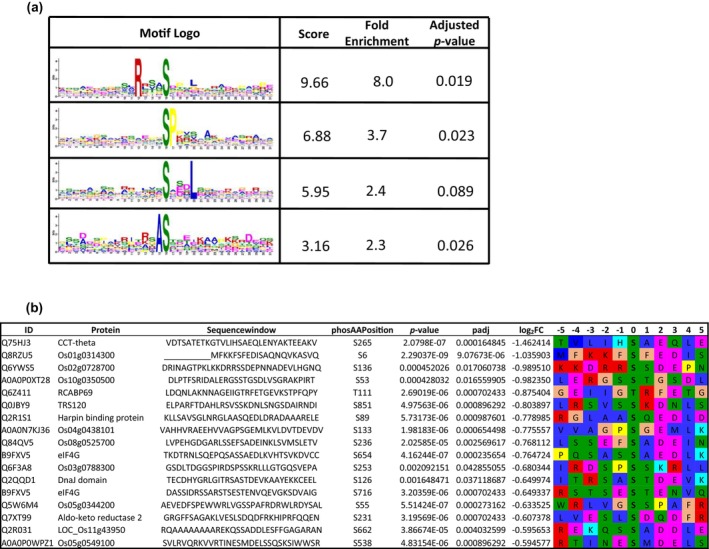
Phosphorylation motif of SnRK1 in rice. (a) Motif enrichment in significantly downregulated phosphosites (*p* adj. < 0.055) in the *snrk1bc* mutant using the MoMo tool with the Motif‐X algorithm (*p* value ≤ 0.01, at least 10 occurrences). (b) Sequence motifs in the selected phosphopeptides (*p* adj. < 0.055; log_2_FC < 0.585) in the *snrk1bc* mutant.

To identify possible SnRK1‐dependent phosphorylation motifs, we performed motif analysis on the significantly downregulated phosphosites (*p* adj. < 0.055) in the *snrk1bc* mutant using the MoMo tool with the Motif‐X algorithm (Cheng et al. 2019). This analysis revealed serine as the predominant residue targeted by SnRK1 (Figure 6a,b). Further, significant enrichment of phosphorylation motifs was seen, particularly those with a P residue at position P + 1 as a classical SP motif, which is characteristic of SnRK1 phosphorylation sites (Cho et al. 2016; Hu et al. 2022; Hu et al. 2024; Nukarinen et al. 2016). Other motifs identified in our dataset include RxxxS (arginine at position P‐4) and SxxxL (leucine at position P + 4) (Figure 6a), which align with previously reported SnRK1 target motifs characterized by basic residues (R, K, or H) at position P‐4 or hydrophobic residues (M, L, V, I, or F) at position P + 4 (van Leene et al. 2022). We also identified a new motif consisting of an alanine (A) at the P‐1 position (Figure 6a). Overall, this analysis shows that SnRK1 phosphorylation motifs are generally conserved between Arabidopsis and rice and share similarities with AMPK phosphorylation motifs, RxxxS and SxxxL (Hardie et al. 2016).

## 3 Discussion

Our integrative phenotypic and omics analyses indicate a key role of SnRK1 signaling in regulating growth and adaptive responses in rice. Rice *snrk1* mutants accumulated lower biomass in the seedlings and mature plants and produced a lower number of seeds per panicles (Figure 1). Notably, growth retardation was stronger in the *snrk1bc* mutant at the early vegetative stage represented by 9‐day‐old seedlings. However, based on the phenotype of plants at maturity, the *snrk1a* mutant showed a greater retardation in the overall growth of shoot and root. These results indicate tissue specificity and functional specialization of *OsSnRK1α* genes. *OsSnRK1α*A is broadly expressed in vegetative and reproductive tissues, supporting a general role in growth and sugar signaling (Takano et al. 1999; Lu et al. 2007), whereas *OsSnRK1α*B and *OsSnRK1α*C are predominantly expressed in developing seeds, with *OsSnRK1α*C showing much higher expression and a strong association with starch accumulation during grain filling (Kanegae et al. 2005). Earlier, Lu et al. (2007) reported retarded germination and shorter shoot and root lengths in 10‐day‐old seedlings of the *ossnrk1a* mutant. However, the role of other *OsSnRK1α* homologs (*OsSnRK1αB* and *OsSnRK1αC*) and the role of SnRK1 in other developmental stages (e.g.,*adult growth*) are not explored well. Concurring with Lu et al., we found slower elongation of shoot and root from the germinating *snrk1* seeds, with stronger retardation observed in the *snrk1bc* mutant (Figure S10). Next, growth retardation and reduced seed set in our *snrk1a* and *snrk1bc* mutants phenocopy rice plants overexpressing *OsSnRK1αA* that also showed decreased shoot and root biomass and lower seed set (Filipe et al. 2018). Taken together, these findings suggest that balanced SnRK1 signaling is critical for proper growth and development, and disturbance in the SnRK1 signaling network leads to growth defects.

The transcriptome of 9‐day‐old seedlings provided a mechanistic basis of growth defects in normal conditions, as it showed upregulation of catabolic processes and stress responses and downregulation of growth processes (Figure 2), mimicking the starvation response. Wang et al. (2021) also found upregulation of starvation‐triggered genes during the energy‐abundant state in rice *snrk1* mutants. Growth processes during energy sufficiency are generally regulated by TOR signaling, which is antagonistic to SnRK1. Thus, it is not clear how starvation‐triggered processes are upregulated, and TOR‐dependent processes are repressed in *snrk1* mutants during energy sufficiency. Nevertheless, these observations underscore the role of SnRK1 in controlling growth and development even in energy‐sufficient (normal) growth conditions. Further, we observed a greater transcriptomic disturbance in *snrk1bc* seedlings than in *snrk1a* seedlings, correlating with the greater phenotypic defect in *snrk1bc* seedlings in normal conditions. However, upregulation of stress‐related processes, including the upregulation of *OsPR3*, *OsPR4*, and *OsPR5*, does not lead to disease resistance in *snrk1* mutants. In fact, susceptibility to blast fungus is enhanced in *snrk1* mutants. Previous studies have reported that *snrk1* mutants show enhanced susceptibility to pathogens and overexpression of *SnRK1A* leads to resistance (Cao et al. 2024; Filipe et al. 2018; Kim et al. 2015; Seo et al. 2011). Therefore, SnRK1 signaling is critical for disease resistance. Concurring with that, a recent study showed SnRK1‐mediated regulation of plant immunity in Arabidopsis through phosphorylation of NPR1 (Chen et al. 2025), a central regulator of salicylic acid‐induced defense response.

As expected, *snrk1* seedlings showed growth defects in the starvation state. Increased yellowing in the culm was observed in both *snrk1* mutants, whereas reduced biomass (shoot and root) was noted in *snrk1bc* seedlings under starvation (Figures 1 and S2–S4). Accordingly, total chlorophyll content was significantly lower in both *snrk1* mutants, and severe phenotypic aberrations were observed in *snrk1bc* seedlings (Figure 1). These observations further point to the functional specialization of *OsSnRK1α* genes and indicate a more prominent role of *OsSnRK1αB* and *OsSnRK1αC* in seedling development and its starvation response. Starvation is a major inducer of SnRK1 signaling (Baena‐González et al. 2007; Henninger et al. 2022; Nukarinen et al. 2016; Pedrotti et al. 2018; Wang et al. 2021). Accordingly, the transcriptome of *snrk1* seedlings under starvation showed a larger set of deregulated genes than in the normal condition and corroborated with the severe defect in the phenotype of *snrk1bc* seedlings (Figures 2 and 3). Interestingly, the normal growth response of the *snrk1bc* mutant mirrored the starvation response of WT as stress and catabolic processes were upregulated in *snrk1bc* in normal condition while being downregulated during starvation (Figure S11). Starvation generally triggers catabolic and stress‐related processes as observed in the WT (Figure 3). Deregulation of these processes in *snrk1bc* suggests that the starvation response in rice seedlings is mostly controlled by OsSnRK1B and OsSnRK1C.

Proteomics and phosphoproteomics provided further validation of the deregulation of SnRK1 signaling in the *snrk1bc* mutant by showing upregulation of peptides and phosphopeptides associated with ribosome biogenesis (Figure 5). The upregulation of this TOR‐dependent process aligns with the current understanding that SnRK1 is required to check TOR activity during starvation (Baena‐González and Hanson 2017; Dobrenel et al. 2016). The downregulated phosphopeptides in *snrk1bc* represent phosphorylation targets of SnRK1. These include core enzymes in the energy generation process, aligning with the consensus that SnRK1 is the hub of metabolic reprogramming in response to starvation (van Leene et al. 2022; Hu et al. 2022). The presence of two proteins functioning in vesicle‐mediated transport (VSP9a and TRS120) among downregulated phosphopeptides implicates SnRK1 in intracellular trafficking and corroborates the study in Arabidopsis that also found a relation of SnRK1 with multiple proteins functioning in protein trafficking, including AtVPS9a (van Leene et al. 2022). Our findings together with those of van Leene et al. align with the emerging evidence in mammals that implicates SnRK1/AMPK in controlling endocytic membrane traffic and ER stress (Chauhan et al. 2020; Rahmani et al. 2019). We also found the downregulation of phosphosites of the key proteins in ethylene signaling: OsEIN2 (Q0D8I9) and OsERF46 (Q6H5V3) (Table S12), which are positive regulators of ethylene signaling and play a central role in development and stress response (Müller and Munné‐Bosch 2015; Jun et al. 2004). Next, three heat shock proteins (Q2QU06, Q2R031, and Q84Q77) and Chaperonin Containing TCP1 Subunit 8 (Q75HJ3; CCT‐theta) were downregulated, indicating a role for SnRK1 in regulating chaperone protein functions. Further, phosphorylation of eIF4G (B9FXV5) was downregulated in the *snrk1bc* mutant. eIF4G is essential for rice tungro virus to manifest disease in susceptible rice cultivars and a mutation in this gene leads to virus resistance (Lee et al. 2010; Macovei et al. 2018). Finally, OsWRKY94 (Q2QMN4) and the proton pump, OVP1 (Q67WN5) were downregulated in the phosphoproteome of the *snrk1bc* mutant. These proteins play crucial roles in cold and salinity tolerance (Chen et al. 2018; Zhang et al. 2011). In summary, several stress‐related phosphopeptides, most of which also play important roles in developmental processes, are downregulated in the *snrk1bc* mutant, indicating a pivotal role of SnRK1 in controlling stress and developmental processes.

We propose the true targets of SnRK1 by searching the literature for SnRK1 targets and checking the phosphorylation motif of the downregulated phosphosites. Earlier, Hu et al. (2022) carried out phosphoproteomics using *snrk1a* mutant to study SnRK1‐mediated transport of carbohydrates from sheath to panicles and predicted several SnRK1 targets in rice based on the parallel reaction monitoring approach of mass spectrometry. We found 9 phosphosites representing seven proteins in our dataset that were predicted to be SnRK1 targets by Hu et al. (Figure S12). One of them, Os04g0679400 protein, contained the classical SnRK1 target motif (SP), raising the confidence of the prediction. VPS9a showed two phosphosites, one of which contained the RxxS motif described as a SnRK1 target in Arabidopsis and rice (van Leene et al. 2022; Nukarinen et al. 2016; Hu et al. 2022), and the other contained VxxxxSxxxL that matches the SnRK1 target motif in Arabidopsis, specifically the presence of a hydrophobic residue at P−5 and P + 4 (van Leene et al. 2022). Five other proteins also contained motifs described as putative SnRK1 targets in the literature that included RxxxS, SD, a hydrophobic residue (M, L V, or I) at P−5 or P + 4, and a basic residue (R) at P−3/P−4 (Figure S12).

In conclusion, this study demonstrates that SnRK1 signaling is essential for integrating energy status in growth and stress responses in rice. Loss of SnRK1 function disrupts transcriptional and posttranslational regulation of metabolic and defense pathways, leading to enhanced disease susceptibility and growth defects. Our findings lead to a potentially novel role for SnRK1 in regulating membrane trafficking during starvation, as indicated by altered phosphorylation of key vesicle‐mediated transport proteins. In addition, novel candidates of SnRK1 were identified through phosphoproteomic analysis; however, their direct phosphorylation by SnRK1 remains to be experimentally validated. Overall, our findings show that SnRK1 signaling is critical for plant adaptation and development under both energy‐sufficient and energy‐deficient conditions. Further, the transcriptomics, proteomics and phosphoproteomics provide a rich resource for further studies focusing on understanding the SnRK1 signaling network in rice.

## 4 Experimental Procedures

### 4.1 Plant Material and Growth Conditions

*snrk1a* and *snrk1bc* mutants were developed by CRISPR/Cas9 targeting of *OsSnRK1α* genes as described earlier (Pathak et al. 2022). For seedling analysis, the homozygous lines of the single mutant of *OsSnRK1αA* (*snrk1a*) and double mutant of *OsSnRK1αB* and *OsSnRK1αC* (*snrk1bc*) along with WT were sterilized and plated on half‐strength MS media (MS½) with 2% sucrose solidified with phytagel (2 g/L). Three to four days after plating, at the S3 stage (when prophyll emerges out of the coleoptile), the seedlings were transferred to glass tubes containing MS½ media without sucrose, solidified with phytagel (1.5 g/L). The seedlings were grown in the growth chamber at 26°C with optimal light (~30 PAR). The light was provided from 6 a.m. to 7 p.m. for a 14 h photoperiod. When the seedlings were at V1 stage (formation of first leaf with collar, ~7 days post‐S3 stage), the seedlings were divided into two groups: Half of them were transferred to complete darkness, mimicking starvation, and the other half were kept under light for two additional days, totaling 9 days of growth in a 14 h photoperiod, representing normal growth. For the phenotyping of mature plants, 7‐day‐old seedlings at V1–V2 stage were transferred to the greenhouse in pots (7 × 7 × 13 cm) filled with commercial potting mix (Promix LP15, Premier Tech Horticulture) consisting of sphagnum peat moss and perlite (9:1). The plants were grown in a randomized block design in the greenhouse and fertilized with iron chelate and Osmocote fertilizer (15 N‐9P‐12 K) and treated with insecticide (abamectin) as needed.

### 4.2 RNA‐seq Analysis

Total RNA from seedlings was isolated using Trizol reagent (Invitrogen Inc.), treated with DNase I, and quantified by Nanodrop 2000 (Thermo Fisher Scientific, United States). Approximately 3 μg per sample (A260/A280 > 1.9) was sent to Novogene Inc. for 150 bp paired‐end directional mRNA (poly A enriched) sequencing, generating 7–9 GB raw data per sample on Illumina HiSeq. Two biological replicates of each genotype/treatment consisting of two to five seedlings each were used. Clean sequences were mapped against *Oryza sativa* japonica Group reference genome IRGSP‐1.0 (EnsemblPlants release, accession GCA_001433935.1; Kawahara et al. 2013) and differentially expressed genes (DEGs) identified using DESeq2 using a threshold of adjusted *p* value (*p* adj.) ≤ 0.05.

### 4.3 Chlorophyll Content

Based on the method described by Pedrotti et al. (2018), 9‐day‐old seedlings (bulked) were frozen in liquid nitrogen. One‐hundred milligrams of frozen tissue was ground in 1 mL of methanol homogenized using a Mixer Mill (MM400; Retsch) and incubated at 60°C for 30 min followed by an additional 10 min at room temperature (RT). The extract was clarified by centrifugation on a benchtop centrifuge and 1:10 dilution of the supernatant was used for measuring absorbance at 650 and 665 nm in a spectrophotometer for chlorophyll a (Chla) and chlorophyll b (Chlb), respectively. Total chlorophyll (mg chlorophyll [Chla + Chlb]/mL extract) was calculated as $\text{total chlorophyll}=A_{\left\{650\right\}}\times 0.025+A_{\left\{665\right\}}\times 0.005$.

### 4.4 Protein Extraction

Snap‐frozen seedlings (bulked) were ground in liquid nitrogen, and 0.5 g of the powdered tissue was transferred into 2.0‐mL microtubes. The ground tissue was suspended in 0.8 mL of extraction buffer (30% sucrose, 2% SDS, 0.1 M Tris–HCl [pH 8.0], and 5% 2‐mercaptoethanol) supplemented with phosphatase inhibitor cocktail (PhosSTOP tablet [Roche] and 20 μL of phenylmethylsulfonyl fluoride). An equal volume of phenol buffered with Tris–HCl (pH 8.8) was added to the mixture, which was then vigorously shaken for 30 s. The samples were centrifuged at 15,000×*g* for 20 min at 4°C, and the upper phenol phase was transferred to fresh microtubes. The remaining tissue was re‐extracted with an additional 0.8 mL of buffered phenol and 0.8 mL of extraction buffer, and the phenol phase was combined with the first extraction. The pooled phenol phase was used to precipitate protein with four volumes of cold methanol containing 100‐mM ammonium acetate, thoroughly mixed and placed at −20°C for 30 min. The samples were centrifuged at 15,000×*g* for 20 min at 4°C, and the supernatant was carefully discarded, and the protein pellet was washed with 1 mL of cold 80% acetone, vortexed, and centrifuged at 15,000×*g* for 10 min at 4°C. This washing was repeated twice, and the washed protein pellet was used for proteomic and phosphoproteomic analysis.

### 4.5 LC‐MS/MS for Discovery Proteomics and Phosphoproteomics

Protein samples were processed at the UAMS Proteomics Core Facility (https://idearesourceproteomics.org/). Total protein from each sample was reduced, alkylated, and purified by chloroform/methanol extraction prior to digestion with sequencing grade modified trypsin/LysC. Peptides were labeled with TMT 10‐plex isobaric label reagent set (Thermo Scientific), and phosphopeptides were enriched using TiO_2_ and Fe‐NTA kits. Enriched and unenriched labeled peptides were fractionated by high‐pH reversed‐phase chromatography and further separated on an XSelect CSH C18 2.5‐um resin (Waters) using an UltiMate 3000 RSLCnano system (Thermo Scientific). MS analysis was performed on an Orbitrap Eclipse Tribrid mass spectrometer (Thermo Scientific) using multinotch MS3 parameters. Proteins were identified and reporter ions quantified by searching the UniprotKB database restricted to *Oryza sativa japonica* using MaxQuant (Max Planck Institute, ver. 2.1.4.0) (Tyanova et al. 2016) with FDR < 0.01. Protein IDs were assigned using the Protein Prophet algorithm (Nesvizhskii et al. 2003). TMT reporter ion intensities from unenriched samples were used to assess changes in total protein abundance, whereas phospho Ser/Thr/Tyr (STY) modifications were identified and quantified from the samples enriched for phosphorylated peptides. Following database search, MS3 reporter ion intensities were normalized using ProteiNorm and variance stabilizing normalization (Graw et al. 2020; Huber et al. 2002). Differential abundance analysis was performed using the limma package with empirical Bayes, smoothing to the standard errors (Ritchie et al. 2015). For phosphopeptides, only sites with localization probability > 75% were considered.

### 4.6 Data Analysis

Differentially expressed genes/proteins with an adjusted *p* value (*p* adj.) of ≤ 0.05 were used to select the significant genes and proteins from the data. ggplot and VennDiagram in R were used to generate volcano plots and Venn diagrams. Cluster 3.0 (de Hoon et al. 2004) was used for clustering the differentially expressed genes using hierarchical clustering and Pearson correlation method. Java Treeview (Saldanha 2004) was used to generate heat maps of the gene clusters. Gene ontology analysis and network analysis were done using String software (Szklarczyk et al. 2019), and the output data were visualized using Cytoscape 3.10.2 (Shannon et al. 2003). Motif analysis was done using the MoMo tool with the Motif‐X algorithm (Cheng et al. 2019) using a significance threshold of *p* ≤ 0.01 and a minimum of 10 occurrences per motif.

### 4.7 Leaf Blast Disease Assays

The *Magnaporthe oryzae* strain Guy11 was grown on 0.8% agar for 10–14 days to allow sufficient sporulation. Once the fungi sporulated, 2 mL of 0.02% gelatin was pipetted onto the plate, and a spatula was used to scrape the spores into the solution. The spores were filtered using one layer of miracloth and collected in 1.5‐mL Eppendorf tubes. The tubes were centrifuged at 5000×*g* for 5 min. The supernatant was removed, and the spores were resuspended in 1 mL 0.02% gelatin. The spores were diluted and counted using a hemocytometer and diluted to a final concentration of 1 × 10^5^ spores per mL. This diluted suspension of spores was used for the disease assay as described below. For leaf spotting, the youngest, fully expanded leaf of 3‐ to 4‐week‐old plants was cut and placed on 0.8% agar media (adaxial side up). Each leaf was inoculated with five drops of 20 μL of spore/gelatin solution and incubated for 5 days. For the leaf spray, 9‐day‐old seedlings grown in MS½ media in a 14 h photoperiod were used. The fully expanded leaf of the seedling was cut and placed on 0.8% agar and inoculated with the spore suspension (described above) using an airbrush sprayer inside a fume hood to ensure uniform coverage. The leaves were placed on the agar plate and incubated for 5 days. For both assays, the infected leaves were scanned using a computer scanner, and the area of each lesion was calculated using auto threshold MaxEntropy of the ImageJ program (Schneider et al. 2012). Differences in lesion areas between genotypes were determined by the Tukey–Kramer test (HSD) using JMP Statistical Discovery 17 from SAS (Version 13.2.1) with the significance threshold of *p* < 0.05.

## Author Contributions

V.S. and K.M.J. designed the project. M.C.F.‐B., K.M.J., and V.S. analyzed the data. C.M. prepared samples for RNA‐seq and proteomics and carried out the initial omics analysis. M.C.F.‐B. and V.S. wrote the paper.

## Conflicts of Interest

The authors declare no conflicts of interest.

## Peer Review

The peer review history for this article is available in the Supporting Information for this article.

## Supporting information


**Data S1:** Peer Review.

**Data S2:** Protein sequence alignments of the kinase subunits of the predicted mutant sequences with the wildtype (WT) of OsSnRK1Aα, OsSnRK1Bα, and OsSnRK1Cα.
**Data S3:** Protein sequence alignments of the kinase subunits of the predicted mutant sequences with the wildtype (WT) of OsSnRK1Aα, OsSnRK1Bα, and OsSnRK1Cα.

**Figure S1:** Gene expression analysis of *OsSnRK1α* genes in rice *snrk1* mutants. Gene expression of *OsSnRK1A*, *OsSnRK1B*, and *OsSnRK1C* genes determined by real‐time qPCR and normalized against *OsUBQ2* gene in 9‐d‐old seedlings of WT, *snrk1a*, *snrk1bc* mutants grown in 14 h photoperiod on MS½ media. The graph shows expression relative to the value for gene in WT. Mean of 2–3 biological replicates are shown with ± standard deviation (SD).
**Figure S2:** Phenotypic characterization of *snrk1* mutants under normal and starvation conditions. (a–d) Boxplots showing shoot length (a), root length (b), shoot biomass (c), and root biomass (d) of 9‐day‐old WT, *snrk1a*, and *snrk1bc* seedlings grown under normal (14‐h light/10‐h dark) conditions. (e–f) Boxplots showing shoot length (e), root length (f), shoot biomass (g), and root biomass (h) of seedlings grown for 7 days under normal condition and then exposed to 48 h of continuous darkness (starvation). Different letters indicate significant differences (*p* < 0.05) determined by one‐way ANOVA followed by Tukey's HSD test on data collected from 10 to 24 seedlings. Error bars represent standard deviation (SD).
**Figure S3:** Starvation‐induced phenotype. (a) Representative images of seedlings of WT, *snrk1a*, and *snrk1bc* after 48 h of continuous darkness (starvation) imposed on 7‐d‐old seedlings. (b‐c) close‐up of the culm at the first leaf collar (b) and fully expanded leaf (c).
**Figure S4:** Phenotypic characterization of *snrk1a.2* and *snrk1bc.2* (second alleles of *ossnrk1αa* and *ossnrk1αbc*). (a‐d) Seedling phenotype and biomass of 9‐d‐old seedlings grown on half‐strength MS medium under 14‐h photoperiod (normal condition) or exposed to 48 h of continuous darkness after 7d of normal growth (starvation). (e–f) Chlorophyll content in 9‐d‐old seedlings under normal or starvation condition. (g–l) Plants at maturity in greenhouse conditions. Representative shoot and root images (g‐h), shoot and root biomass (i‐j), and reproductive traits (k‐l). (m‐n) Disease response of detached leaves following inoculation with *M. oryzae* strain Guy11. YT16 is a susceptible rice. Significant differences (*p* < 0.05) by one‐way ANOVA followed by Tukey's HSD test is shown by small betters. Error bars represent standard deviation (SD). *n* = 16 (a‐f), *n* = 20 (g‐l), *n* = 18 (m‐n).
**Figure S5:** Principal component analysis (PCA) of transcriptomic profiles of *snrk1* mutants and WT under normal or starvation conditions. PCA plots of RNA‐seq data (normalized gene count) from 9‐d‐old WT, *snrk1a*, and *snrk1bc* seedlings grown under normal condition (14‐h photoperiod) or subjected to 48 h of continuous darkness after 7 days of normal growth (starvation condition). PCA plots were generated on iDEP web‐based tool (Ge et al., 2018).
**Figure S6:** Differential gene expression in WT and *snrk1* mutants under starvation compared to normal growth. Volcano plots showing transcriptomic changes in (a) WT, (b) *snrk1a*, and (c) *snrk1bc* seedlings after 48 h of continuous darkness (starvation) versus those grown in normal condition. Differentially expressed genes (DEGs) were identified based on P‐adj. < 0.05 and |log_2_FC| > 0.
**Figure S7:** Gene expression analysis of Os*SnRK1α* subunit genes and rice homologs of Arabidopsis dark‐induced 6 (*DIN6*). qPCR analysis on the rice *OsSnRK1α* paralogs (A, B, C) and the rice homologs of Arabidopsis *DARK INDUCIBLE 6/ASPARAGINE SYNTHASE 1* (*DIN6/ASN1*) (*OsASN1* and *OsASN2*) in 9‐d‐old seedlings of WT Kitaake seedlings grown in 14 h photoperiod (normal) or exposed to 2 days of continuous darkness after 7 days of normal growth (starvation). Gene expression was normalized against *OsUBQ2* gene (Os02g0161900). Average of 2–3 samples (biological replicates) was plotted with standard deviation shown as error bars.
**Figure S8:** Protein interaction network associated with differentially‐abundant phosphosites in *snrk1bc* mutant under extended darkness. STRING‐based network analysis of significantly abundant phosphosites in *snrk1bc* seedlings under starvation (p‐adj. < 0.055; |log_2_ fold change| > 0). Nodes represent proteins and edges represent known or predicted protein–protein interactions. Nodes are color‐coded by log_2_ fold change: red indicates proteins associated with upregulated phosphosites and blue indicates proteins associated with downregulated phosphosites. Selected networks associated with gene ontology (GO) terms are circled.
**Figure S9:** Phosphorylation status of conserved TOR‐S6K targets in *snrk1bc*. Enhanced phosphorylation (log2 fold change) of ribosomal protein S6 (RPS6) and eukaryotic translation initiation factors (eIFs) in *snrk1bc* compared to WT under starvation. Significance (*p*‐value) is indicated as * or **. The rest of the phosphosites show non‐significant fold‐change.
**Figure S10:** Seed germination on half‐strength MS media. (a) percent germination based on number of seeds showing emergence of coleoptile after 3 days on the media at 28oC in 14 h photoperiod (*n* = 70). (b) Representative images of germinating seeds of WT and *snrk1* mutants on the media. Note slower rate of shoot and root elongation in *snrk1* mutants, although percent germination is not significantly different (*p* < 0.05) by one‐way ANOVA followed by Tukey's HSD test.
**Figure S11:** Transcriptomic profile of *snrk1bc* under normal condition mirrors the transcriptomic profile of WT under starvation. Hierarchical clustering of differentially expressed genes in WT under starvation (WT*_*starvation vs. WT*_*normal) and *snrk1bc* mutant under normal condition (*snrk1bc_*normal vs. WT_normal). Genes were clustered based on fold‐change, with red indicating upregulation and blue indicating downregulation. Associated gene ontology (GO) terms or functional keywords are annotated.
**Figure S12:** Conserved SnRK1 target motifs identified in rice. Phosphosites corresponding to proteins found in our dataset and previously reported in the literature as SnRK1 targets in rice. Columns include the UniProt protein ID, gene name, the 15‐amino‐acid phosphosite sequence window (centered on the phosphorylated residue), position of the phospho‐acceptor (S or T) within the full‐length protein, nominal p‐value, adjusted p‐value (p‐adj.), and log_2_ old change (log_2_FC) comparing *snrk1bc* to WT.

**Table S1:** Differentially expressed genes in *snrk1a* mutant compared to WT under normal condition.
**Table S2:** Differentially expressed genes in *snrk1bc* mutant compared to WT under normal condition.
**Table S3:** Enriched GO terms in *snrk1a* mutant compared to WT under normal condition.
**Table S4:** Enriched GO terms in *snrk1bc* mutant compared to WT grown under normal condition.
**Table S5:** Differentially expressed genes in wildtype (WT) exposed to starvation condition consisting of extended darkness in comparison to WT under normal condition (14‐h photoperiod).
**Table S6:** Differentially expressed genes in *snrk1a* mutant exposed to starvation condition consisting of extended darkness in comparison to *snrk1a* under normal condition (14‐h photoperiod).
**Table S7:** Differentially expressed genes in *snrk1bc* mutant exposed to starvation condition consisting of extended darkness in comparison to *snrk1bc* under normal condition (14‐h photoperiod).
**Table S8:** Enriched GO terms in WT under 2 days of continuous darkness (starvation).
**Table S9:** Enriched GO terms in *snrk1a* mutant under 2 days of continuous darkness (starvation).
**Table S10:** Enriched GO terms in *snrk1bc* mutant under 2 days of continuous darkness (starvation).
**Table S11:** Differentially abundant peptides in snrk1bc mutant compared to WT.
**Table S12:** Differentially‐abundant phosphopetides in *snrk1bc* mutant compared to WT under 2 days of continuous darkness.
**Table 13:** Enrichment analysis of differentially expressed phosphopeptides in snrk1bc mutant.

## Data Availability

The proteomic and phosphoproteomic data are available from the corresponding author. The NCBI (SRA) accession number associated with this study is SUB15675617.
