# Supplementary material for: Integrative Transcriptomic and Phosphoproteomic Analysis Reveals Key Components of the SnRK1 Signaling Network in Rice
Source: Plant Direct. 2025 Nov 17;9(11):e70120. doi: 10.1002/pld3.70120 (PMC12623061; doi:10.1002/pld3.70120)
Supplement: Supplementary file 2 — Data S2: Protein sequence alignments of the kinase subunits of the predicted mutant sequences with the wildtype (WT) of OsSnRK1Aα, OsSnRK1Bα, and OsSnRK1Cα. Data S3: Protein sequence alignments of the kinase subunits of the predicted mutant sequences with the wildtype (WT) of OsSnRK1Aα, OsSnRK1Bα, and OsSnRK1Cα. [file PLD3-9-e70120-s001.docx]

**Supplementary Material**

**Integrative transcriptomic and phosphoproteomic analysis reveals key components of SnRK1 signaling network in rice**

**Maria C. Faria-Bates^1^, Chandan Maurya^1^, Mohammed Jamsheer K^2,3^, and Vibha Srivastava^1^**

^1^Dept. of Crop, Soil & Environmental Sciences, University of Arkansas System Division of

Agriculture, Fayetteville, AR 72701, USA; ^2^Amity Institute of Genome Engineering, Amity

University Uttar Pradesh, Sector-125, Noida-201303, India; ^3^Dept. of Immunobiology, University

of Lausanne, Epalinges, Switzerland.

*Corresponding author

Email: [vibhas@uark.edu](mailto:vibhas@uark.edu)

**Supplementary File S1:** Protein sequence alignments of the kinase subunits of the predicted mutant sequences with the wildtype (WT) of OsSnRK1Aα, OsSnRK1Bα, and OsSnRK1Cα.

Kinase domain, Activation loop, and UBA region are highlighted, ATP binding site is shown in bold, and phosphorylation site in activation loop is highlighted in red

1. **Alignment of the predicted OsSnRK1Aα protein sequence in the *snrk1a* mutant with WT**

**OsSnRK1αA (LOC_Os5g45420/ Os05g0530500)**

WT MEGAGRDGNPLGGYRIGKT**LGIGSFGKV**KIAEHILTGHKVAIKILNRRKIKSMEMEEKVK 60

snrk1a MEGAGRDGNPLGGYRIGKT**LGIGSFGKV**KIAEHILTGHKVAIKILNRRKIKSMEMEEKVK 60

************************************************************

WT REIKILRLFMHPHIIRLYEVIDTPADIYVVMEYVKSGELFDYIVEKGRLQEEEARRFFQQ 120

snrk1a REIKILRLFMHPHIIRLYEVIDTPADIYVVMEYVKSGELFDYIVEKGRLQEEEARRFFQQ 120

************************************************************

WT IISGVEYCHRNMVVHRDLKPENLLLDSKCNVKIADFGLSNVMRDGHFLKTSCGSPNYAAP 180

snrk1a IISGVEYCHRNMVVHRDLKPENLLLDSKCNVKIADFGLSNVMRDGHFLKTSCGSPNYAAP 180

************************************************************

WT EVISGKLYAGPEVDVWSCGVILYALLCGTLPFDDENIPNLFKKIKGGIYTLPSHLSPLAR 240

snrk1a EVISGKLYAGPEVDVWSCGVILYALLCGTLHLMTRIFPTFLRK----------------- 223

****************************** : . :*.:::*

WT DLIPRMLVVDPMKRITIREIREHQWFTVGLPRYLAVPPPDTAQQVKKLDDETLNDVINMG 300

snrk1a ------------------------------------------------------------ 223

WT FDKNQLIESLHKRLQNEATVAYYLLLDNRLRTTSGYLGAEFHESMESSLAQVTPAETPNS 360

snrk1a ------------------------------------------------------------ 223

WT ATDHRQHGHMESPGFGLRHHFAADRKWALGLQSRAHPREIITEVLKALQELNVCWKKIGH 420

snrk1a ------------------------------------------------------------ 223

WT YNMKCRWSPSFPSHESMMHNNHGFGAESAIIETDDSEKSTHTVKFEIQLYKTRDEKYLLD 480

snrk1a ------------------------------------------------------------ 223

WT LQRVSGPQLLFLDLCSAFLTQLRVL 505

snrk1a ------------------------- 223

1. **Alignments of the predicted OsSnRK1αB and OsSnRK1αC protein sequences in the *snrk1bc* mutant with WT**

**OsSnRK1αB (LOC_Os3g17980/ Os03g0289100)**

snrk1bc MEGNARGGGHSEALKNYNLGRT**LGIGSFGKV**KIAEHKLTGHRVAIKILNRRQMRNMEMEE 60

WT MEGNARGGGHSEALKNYNLGRT**LGIGSFGKV**KIAEHKLTGHRVAIKILNRRQMRNMEMEE 60

************************************************************

snrk1bc KAKREIKILRLFIHPHIIRLYEVIYTPTDIYVVMEYCKFGELFDYIVEKGRLQEDEARRI 120

WT KAKREIKILRLFIHPHIIRLYEVIYTPTDIYVVMEYCKFGELFDYIVEKGRLQEDEARRI 120

************************************************************

snrk1bc FQQIISGVEYCHSGGSS------------------------------------------- 137

WT FQQIISGVEYCHRNMVVHRDLKPENLLLDSKYNVKLADFGLSNVMHDGHFLKTSCGSPNY 180

************ .

snrk1bc ------------------------------------------------------------ 137

WT AAPEVISGKLYAGPEVDVWSCGVILYALLCGTLPFDDENIPNLFKKIKGGIYTLPSHLSA 240

snrk1bc ------------------------------------------------------------ 137

WT LARDLIPRMLVVDPMKRITIREIREHQWFQIRLPRYLAVPPPDTAQQAKMIDEDTLQDVV 300

snrk1bc ------------------------------------------------------------ 137

WT NLGYEKDHVCESLRNRLQNEATVAYYLLLDNRFRATSGYLGADYQESLERNLNRFASSES 360

snrk1bc ------------------------------------------------------------ 137

WT ASSNTRHYLPGSSDPHASGLRPHYPVERKWALGLQSRAQPREIMIEVLKALEDLNVCWKK 420

snrk1bc ------------------------------------------------------------ 137

WT NGQYNMKCRWSVGYPQATDMLDVNHSFVDDSIIMDNGDVNGRLPAVIKFEIQLYKSRDEK 480

snrk1bc ------------------------------ 137

WT YLLDMQRVTGPQLLFLDFCAAFLTKLRVL* 509

**OsSnRK1αC (LOC_Os8g37800/Os8g0484600)**

snrk1bc MLTRTITYCMVSVTHRIHHPSIMNKLFSTAWILRHVLRWFKVKMDGNAKGGGHSEALKNY 60

WT MLTRTITYCMVSVTHRIHHPSIMNKLFSTAWILRHVLRWFKVKMDGNAKGGGHSEALKNY 60

************************************************************

snrk1bc NLGRT**LGIGSFGKV**KIAEHKLTGHRVAIKILNRRQMRNMEMEEKAKREIKILRLFIHPHI 120

WT NLGRT**LGIGSFGKV**KIAEHKLTGHRVAIKILNRRQMRNMEMEEKAKREIKILRLFIHPHI 120

************************************************************

snrk1bc IRLYEVIYTPTDIYVVMEYCKFGELFDYIVEKGRLQEDEARRIFQADYIWG--------- 171

WT IRLYEVIYTPTDIYVVMEYCKFGELFDYIVEKGRLQEDEARRIFQQIISGVEYCHRNMVV 180

*********************************************

snrk1bc ------------------------------------------------------------ 171

WT HRDLKPENLLLDSKYNVKLADFGLSNVMHDGHFLKTSCGSPNYAAPEVISGKLYAGPEVD 240

snrk1bc ------------------------------------------------------------ 171

WT VWSCGVILYALLCGTLPFDDENIPNLFKKIKGGIYTLPSHLSALARDLIPRMLVVDPMKR 300

snrk1bc ------------------------------------------------------------ 171

WT ITIREIREHQWFQIRLPRYLAVPPPDTAQQAKMIDEDTLQDVVNLGYGKDHVCESLRNRL 360

snrk1bc ------------------------------------------------------------ 171

WT QNEATVAYYLLLDNRFRATSGYLGADYQESLERNFNRFASSESASSNTRHYLPGSSDPHA 420

snrk1bc ------------------------------------------------------------ 171

WT SGLRPHYPVERKWALGLQSRAQPREIMIEVLKALQDLNVSWKKNGQYNMKCRWSVGTQAT 480

snrk1bc ------------------------------------------------------------ 171

WT DMLDVNNSFVDDSIIMDNGDVNGRLPAVIKFEIQTRDEKYLLDMQRVTGPQLLFLDFCAD 540

snrk1bc --------- 171

WT FLTKLRVL* 548

**Supplementary File S2:** Protein sequence alignments of the kinase subunits of the predicted mutant sequences with the wildtype (WT) of OsSnRK1Aα, OsSnRK1Bα, and OsSnRK1Cα.

Kinase domain, Activation loop, and UBA region are highlighted, ATP binding site is shown in bold, and phosphorylation site in activation loop is highlighted in red

1. **Alignment of the predicted OsSnRK1Aα protein sequence in the *snrk1a.2* mutant with WT**

**OsSnRK1αA (LOC_Os5g45420/ Os05g0530500)**

snrk1a.2 MEGAGRDGNPLGGYRIGKT**LGIGSFGKV**KIAEHILTGHKVAIKILNRRKIKSMEMEEKVK 60

WT MEGAGRDGNPLGGYRIGKT**LGIGSFGKV**KIAEHILTGHKVAIKILNRRKIKSMEMEEKVK 60

************************************************************

snrk1a.2 REIKILRLFMHPHIIRLYEVIDTPADIYVVMEYVKSGELFDYIVEKGRLQEEEARRFFQQ 120

WT REIKILRLFMHPHIIRLYEVIDTPADIYVVMEYVKSGELFDYIVEKGRLQEEEARRFFQQ 120

************************************************************

snrk1a.2 IISGVEYCHRNMVVHRDLKPENLLLDSKCNVKIADFGLSNVMRDGHFLKTSCGSPNYAAP 180

WT IISGVEYCHRNMVVHRDLKPENLLLDSKCNVKIADFGLSNVMRDGHFLKTSCGSPNYAAP 180

************************************************************

snrk1a.2 EVISGKLYAGPEVDVWSCGVILYALLCGTL-LDDENIPNLFKKIKGGIYTLPSHLSPLAR 239

WT EVISGKLYAGPEVDVWSCGVILYALLCGTLPFDDENIPNLFKKIKGGIYTLPSHLSPLAR 240

****************************** ****************************

snrk1a.2 DLIPRMLVVDPMKRITIREIREHQWFTVGLPRATS------------------------- 274

WT DLIPRMLVVDPMKRITIREIREHQWFTVGLPRYLAVPPPDTAQQVKKLDDETLNDVINMG 300

********************************

snrk1a.2 ------------------------------------------------------------ 274

WT FDKNQLIESLHKRLQNEATVAYYLLLDNRLRTTSGYLGAEFHESMESSLAQVTPAETPNS 360

snrk1a.2 ------------------------------------------------------------ 274

WT ATDHRQHGHMESPGFGLRHHFAADRKWALGLQSRAHPREIITEVLKALQELNVCWKKIGH 420

snrk1a.2 ------------------------------------------------------------ 274

WT YNMKCRWSPSFPSHESMMHNNHGFGAESAIIETDDSEKSTHTVKFEIQLYKTRDEKYLLD 480

snrk1a.2 ------------------------- 274

WT LQRVSGPQLLFLDLCSAFLTQLRVL 505

1. **Alignments of the predicted OsSnRK1αB and OsSnRK1αC protein sequences in the *snrk1bc.2* mutant with WT**

**OsSnRK1αB (LOC_Os3g17980/ Os03g0289100)**

snrk1bc.2 MEGNARGGGHSEALKNYNLGRT**LGIGSFGKV**KIAEHKLTGHRVAIKILNRRQMRNMEMEE 60

WT MEGNARGGGHSEALKNYNLGRT**LGIGSFGKV**KIAEHKLTGHRVAIKILNRRQMRNMEMEE 60

************************************************************

snrk1bc.2 KAKREIKILRLFIHPHIIRLYEVIYTPTDIYVVMEYCKFGELFDYIVEKGRLQEDEARRI 120

WT KAKREIKILRLFIHPHIIRLYEVIYTPTDIYVVMEYCKFGELFDYIVEKGRLQEDEARRI 120

************************************************************

snrk1bc.2 FQQIISGVEYCHRNSGSS------------------------------------------ 138

WT FQQIISGVEYCHRNMVVHRDLKPENLLLDSKYNVKLADFGLSNVMHDGHFLKTSCGSPNY 180

************** .

snrk1bc.2 ------------------------------------------------------------ 138

WT AAPEVISGKLYAGPEVDVWSCGVILYALLCGTLPFDDENIPNLFKKIKGGIYTLPSHLSA 240

snrk1bc.2 ------------------------------------------------------------ 138

WT LARDLIPRMLVVDPMKRITIREIREHQWFQIRLPRYLAVPPPDTAQQAKMIDEDTLQDVV 300

snrk1bc.2 ------------------------------------------------------------ 138

WT NLGYEKDHVCESLRNRLQNEATVAYYLLLDNRFRATSGYLGADYQESLERNLNRFASSES 360

snrk1bc.2 ------------------------------------------------------------ 138

WT ASSNTRHYLPGSSDPHASGLRPHYPVERKWALGLQSRAQPREIMIEVLKALEDLNVCWKK 420

snrk1bc.2 ------------------------------------------------------------ 138

WT NGQYNMKCRWSVGYPQATDMLDVNHSFVDDSIIMDNGDVNGRLPAVIKFEIQLYKSRDEK 480

snrk1bc.2 ------------------------------ 138

WT YLLDMQRVTGPQLLFLDFCAAFLTKLRVL* 509

**OsSnRK1αC (LOC_Os8g37800/ Os8g0484600)**

snrk1bc.2 MLTRTITYCMVSVTHRIHHPSIMNKLFSTAWILRHVLRWFKVKMDGNAKGGGHSEALKNY 60

WT MLTRTITYCMVSVTHRIHHPSIMNKLFSTAWILRHVLRWFKVKMDGNAKGGGHSEALKNY 60

************************************************************

snrk1bc.2 NLGRT**LGIGSFGKV**KIAEHKLTGHRVAIKILNRRQMRNMEMEEKAKREIKILRLFIHPHI 120

WT NLGRT**LGIGSFGKV**KIAEHKLTGHRVAIKILNRRQMRNMEMEEKAKREIKILRLFIHPHI 120

************************************************************

snrk1bc.2 IRLYEVIYTPTDIYVVMEYCKFGELFDYIVEKGRLQEDEARRIFQQIISGVEYCHRNS-- 178

WT IRLYEVIYTPTDIYVVMEYCKFGELFDYIVEKGRLQEDEARRIFQQIISGVEYCHRNMVV 180

*********************************************************

snrk1bc.2 ------------------------------------------------------------ 178

WT HRDLKPENLLLDSKYNVKLADFGLSNVMHDGHFLKTSCGSPNYAAPEVISGKLYAGPEVD 240

snrk1bc.2 ------------------------------------------------------------ 178

WT VWSCGVILYALLCGTLPFDDENIPNLFKKIKGGIYTLPSHLSALARDLIPRMLVVDPMKR 300

snrk1bc.2 ------------------------------------------------------------ 178

WT ITIREIREHQWFQIRLPRYLAVPPPDTAQQAKMIDEDTLQDVVNLGYGKDHVCESLRNRL 360

snrk1bc.2 ------------------------------------------------------------ 178

WT QNEATVAYYLLLDNRFRATSGYLGADYQESLERNFNRFASSESASSNTRHYLPGSSDPHA 420

snrk1bc.2 ------------------------------------------------------------ 178

WT SGLRPHYPVERKWALGLQSRAQPREIMIEVLKALQDLNVSWKKNGQYNMKCRWSVGTQAT 480

snrk1bc.2 ------------------------------------------------------------ 178

WT DMLDVNNSFVDDSIIMDNGDVNGRLPAVIKFEIQTRDEKYLLDMQRVTGPQLLFLDFCAD 540

snrk1bc.2 --------- 178

WT FLTKLRVL* 548

**Supplementary methods:**

**Gene Expression Analysis:** Two micrograms of total RNA were treated with RQ1-RNAse free DNase (Thermofisher Inc.), and one microgram of the DNase-treated RNA was used for cDNA synthesis using PrimeScript RT reagent kit (Takara Bio, CA, USA). The expression analysis was performed using TB green Premix Ex Taq II (Takara Bio, CA, USA) on Bio-Rad CFX 96 C1000 with following conditions: 95°C for 30 sec and 40 cycles of 95°C for 5 sec + 60°C for 30 sec. The product specificity was verified by the melt curve analysis. Rice *UBQ2* gene was used as the internal control. Primer sequences used in this study are given below:

**List of qPCR primers:**

| **Gene** | **Gene ID** | **Sequence (5’ to 3’)** |
| --- | --- | --- |
| *OsSnRK1A* | Os05g0530500 | Forward: GCAGACTTTGGCTTGAGTAATG  Reverse: AACTTCAGGGCCAGCATATAG |
| *OsSnRK1B* | Os08g0484600 | Forward: GCTTGCTGACTTTGGTTTGAG  Reverse: CCTCGGGTCCAGCATATAATTT |
| *OsSnRK1C* | Os03g0289100 | Forward: GCAGACTTTGGCTTGAGTAATG  Reverse: AACTTCAGGGCCAGCATATAG |
| *OsASN1* | Os03g0291500 | Forward: CGGTTACCTCTACTTCCACTTC  Reverse: GTTAGCACGCAGACAGTCATA |
| *OsASN2*  *OsUBQ2* | Os06g0265000  Os02g0161900 | Forward: GGTGGACTGGACTCTTCTTTG  Reverse: GCAGCTCTAAGATCAGGAGAAC  Forward: TGGTCAGTAATCAGCCAGTTTG  Reverse: CAAATACTTGACGAACAGAGGC |

**Supplementary References:**

Ge, S. X., Son, E. W., & Yao, R. (2018). iDEP: an integrated web application for differential expression and pathway analysis of RNA-Seq data. BMC Bioinformatics, 19(1), Article 534. https://doi.org/10.1186/s12859-018-2486-6
