# Supplementary material for: Integrative Transcriptomic and Phosphoproteomic Analysis Reveals Key Components of the SnRK1 Signaling Network in Rice
Source: Plant Direct. 2025 Nov 17;9(11):e70120. doi: 10.1002/pld3.70120 (PMC12623061; doi:10.1002/pld3.70120)
Supplement: Supplementary file 3 — Figure S1: Gene expression analysis of OsSnRK1α genes in rice snrk1 mutants. Gene expression of OsSnRK1A, OsSnRK1B, and OsSnRK1C genes determined by real‐time qPCR and normalized against OsUBQ2 gene in 9‐d‐old seedlings of WT, snrk1a, snrk1bc mutants grown in 14 h photoperiod on MS½ media. The graph shows expression relative to the value for gene in WT. Mean of 2–3 biological replicates are shown with ± standard deviation (SD). Figure S2: Phenotypic characterization of snrk1 mutants under normal and starvation conditions. (a–d) Boxplots showing shoot length (a), root length (b), shoot biomass (c), and root biomass (d) of 9‐day‐old WT, snrk1a, and snrk1bc seedlings grown under normal (14‐h light/10‐h dark) conditions. (e–f) Boxplots showing shoot length (e), root length (f), shoot biomass (g), and root biomass (h) of seedlings grown for 7 days under normal condition and then exposed to 48 h of continuous darkness (starvation). Different letters indicate significant differences (p < 0.05) determined by one‐way ANOVA followed by Tukey's HSD test on data collected from 10 to 24 seedlings. Error bars represent standard deviation (SD). Figure S3: Starvation‐induced phenotype. (a) Representative images of seedlings of WT, snrk1a, and snrk1bc after 48 h of continuous darkness (starvation) imposed on 7‐d‐old seedlings. (b‐c) close‐up of the culm at the first leaf collar (b) and fully expanded leaf (c). Figure S4: Phenotypic characterization of snrk1a.2 and snrk1bc.2 (second alleles of ossnrk1αa and ossnrk1αbc). (a‐d) Seedling phenotype and biomass of 9‐d‐old seedlings grown on half‐strength MS medium under 14‐h photoperiod (normal condition) or exposed to 48 h of continuous darkness after 7d of normal growth (starvation). (e–f) Chlorophyll content in 9‐d‐old seedlings under normal or starvation condition. (g–l) Plants at maturity in greenhouse conditions. Representative shoot and root images (g‐h), shoot and root biomass (i‐j), and reproductive traits (k‐l). (m‐n) Disease re [file PLD3-9-e70120-s002.pdf]

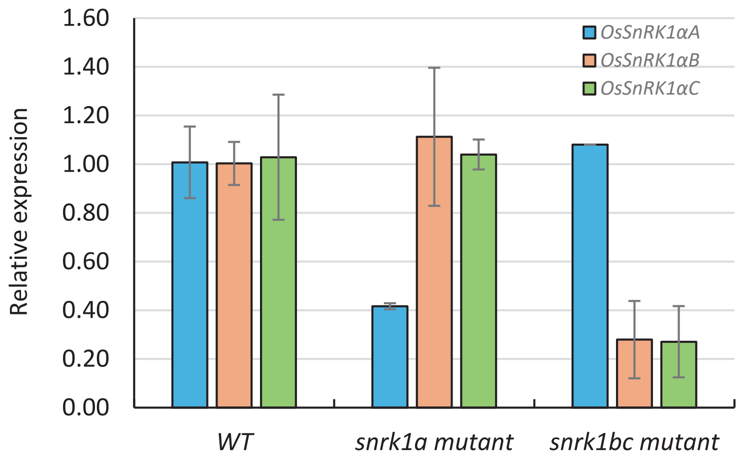

Figure S1: Gene expression analysis of *OsSnRK1α* genes in rice *snrk1* mutants. Gene expression of *OsSnRK1A*, *OsSnRK1B*, and *OsSnRK1C* genes determined by real-time qPCR and normalized against *OsUBQ2* gene in 9-d-old seedlings of WT, *snrk1a*, *snrk1bc* mutants grown in 14 h photoperiod on MS½ media. The graph shows expression relative to the value for gene in WT. Mean of 2 – 3 biological replicates are shown with ± standard deviation (SD).

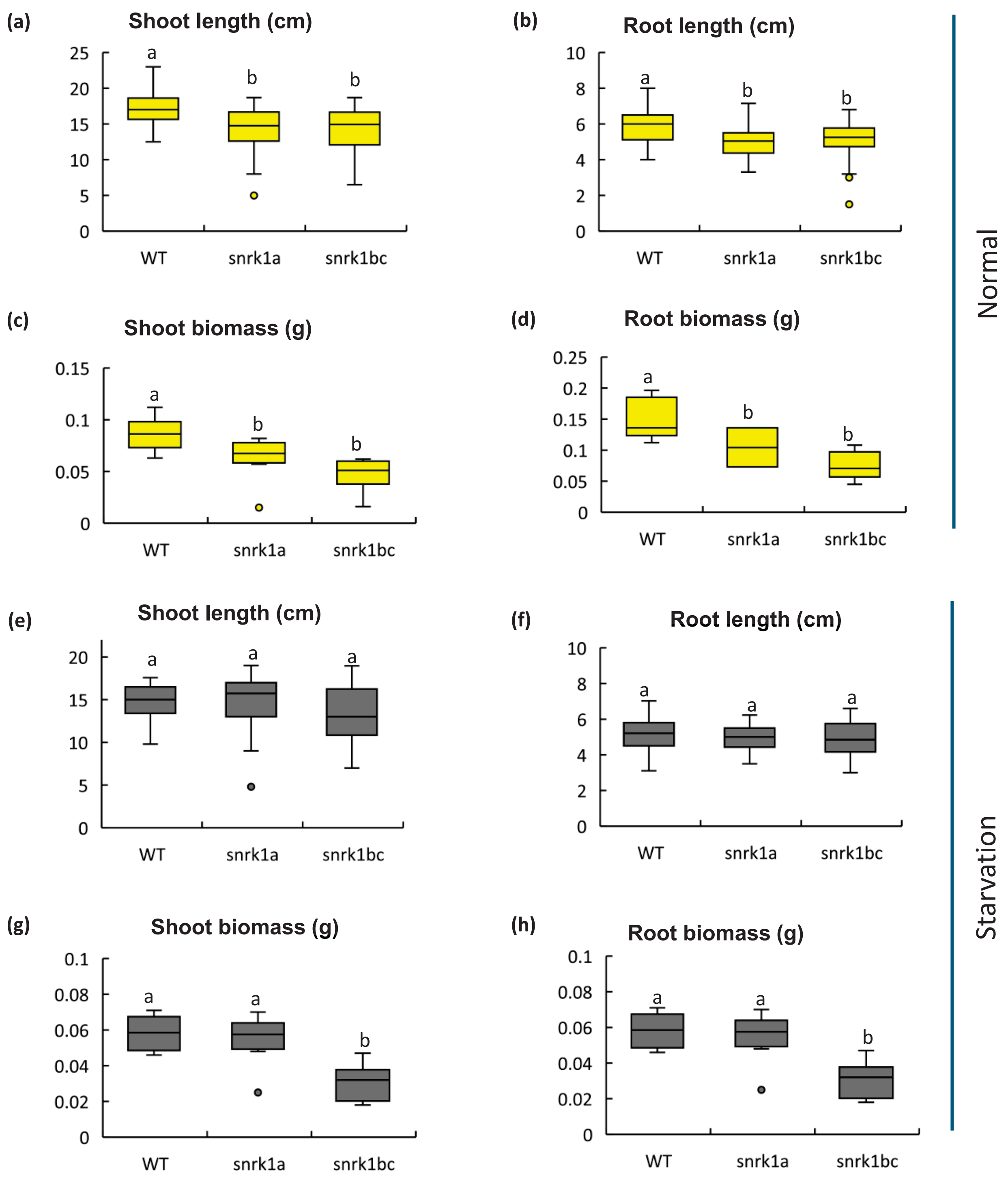

Figure S2: Phenotypic characterization of *snrk1* mutants under normal and starvation conditions. **(a–d)** Boxplots showing shoot length **(a)**, root length **(b)**, shoot biomass **(c)**, and root biomass **(d)** of 9-day-old WT, *snrk1a*, and *snrk1bc* seedlings grown under normal (14-h light/10-h dark) conditions. **(e–f)** Boxplots showing shoot length **(e)**, root length **(f)**, shoot biomass **(g)**, and root biomass **(h)** of seedlings grown for 7 days under normal condition and then exposed to 48 h of continuous darkness (starvation). Different letters indicate significant differences (p<0.05) determined by one-way ANOVA followed by Tukey's HSD test on data collected from 10 to 24 seedlings. Error bars represent standard deviation (SD).

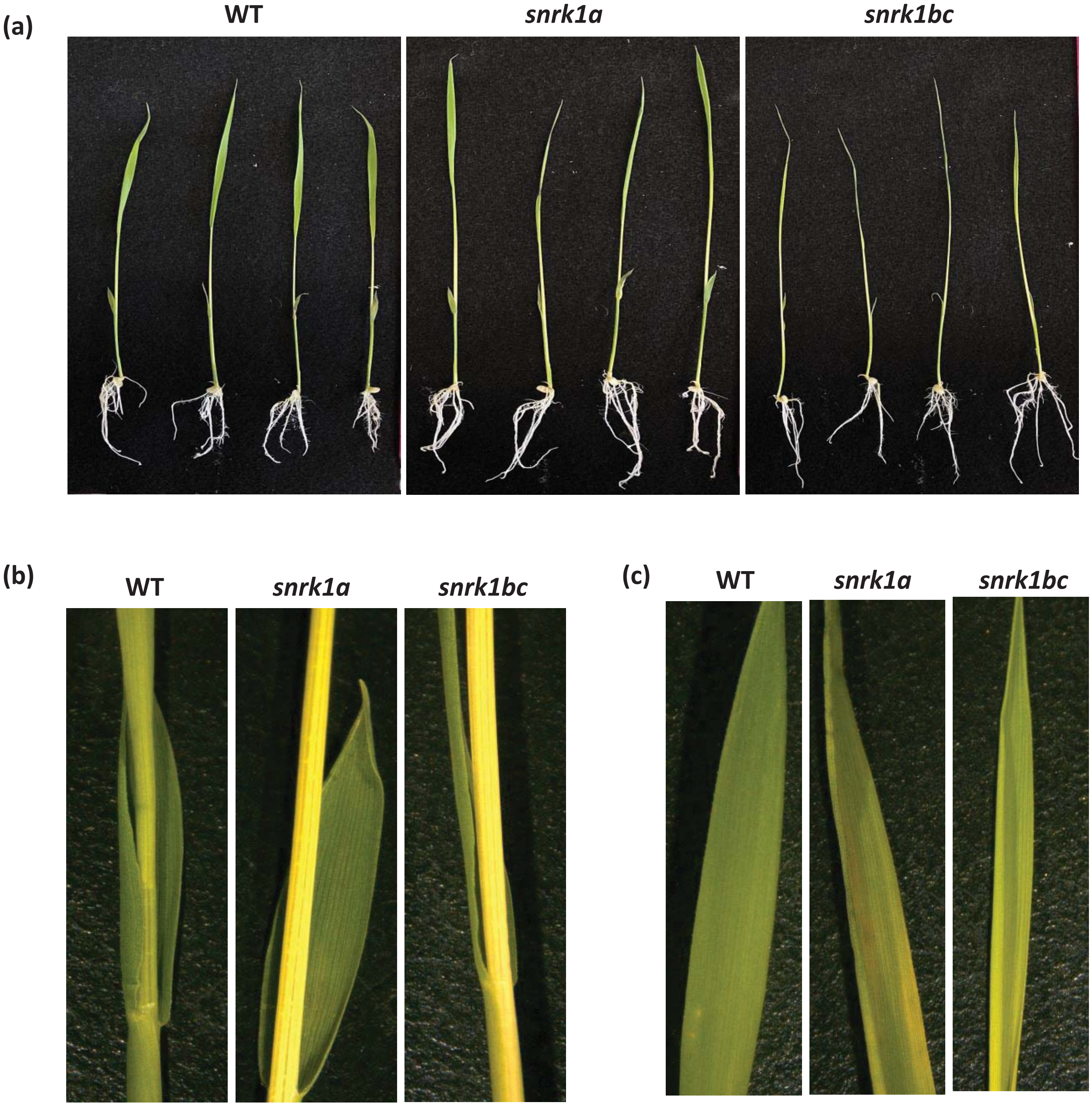

Figure S3. Starvation-induced phenotype. (a) Representative images of seedlings of WT, *snrk1a*, and *snrk1bc* after 48 h of continuous darkness (starvation) imposed on 7-d-old seedlings. (**b-c**) close-up of the culm at the first leaf collar (b) and fully expanded leaf (c).

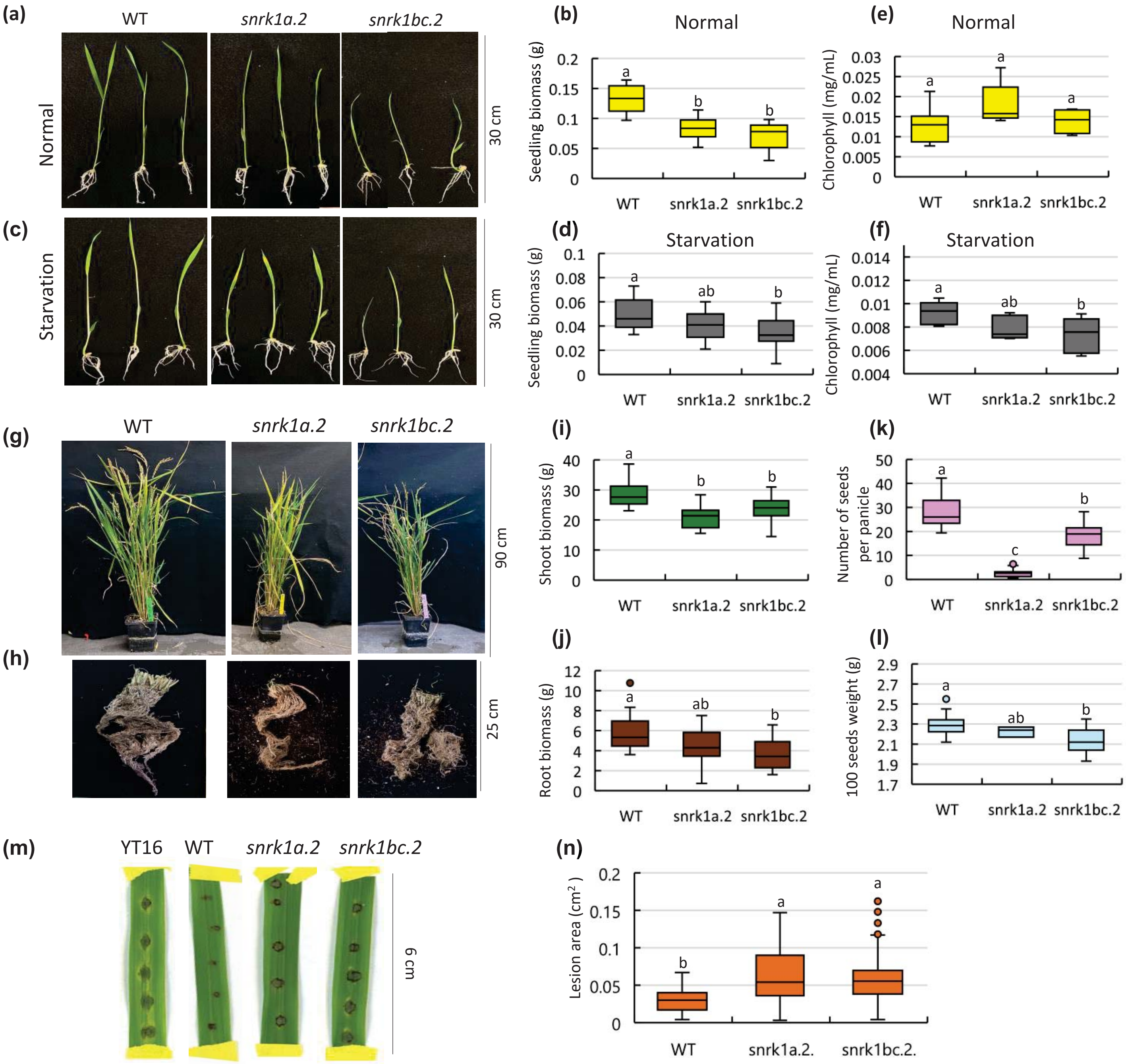

Figure S4: Phenotypic characterization of *snrk1a.2* and *snrk1bc.2* (second alleles of *ossnrk1αa* and *ossnrk1αbc*). **(a-d)** Seedling phenotype and biomass of 9-d-old seedlings grown on half-strength MS medium under 14-h photoperiod (normal condition) or exposed to 48 h of continuous darkness after 7d of normal growth (starvation). **(e–f)** Chlorophyll content in 9-d-old seedlings under normal or starvation condition. **(g–l)** Plants at maturity in greenhouse conditions. Representative shoot and root images (g-h), shoot and root biomass (i-j), and reproductive traits (k-l). **(m-n)** Disease response of detached leaves following inoculation with *M. oryzae* strain Guy11. YT16 is a susceptible rice. Significant differences (p<0.05) by one-way ANOVA followed by Tukey's HSD test is shown by small betters. Error bars represent standard deviation (SD). n= 16 (a-f), n = 20 (g-l), n = 18 (m-n).

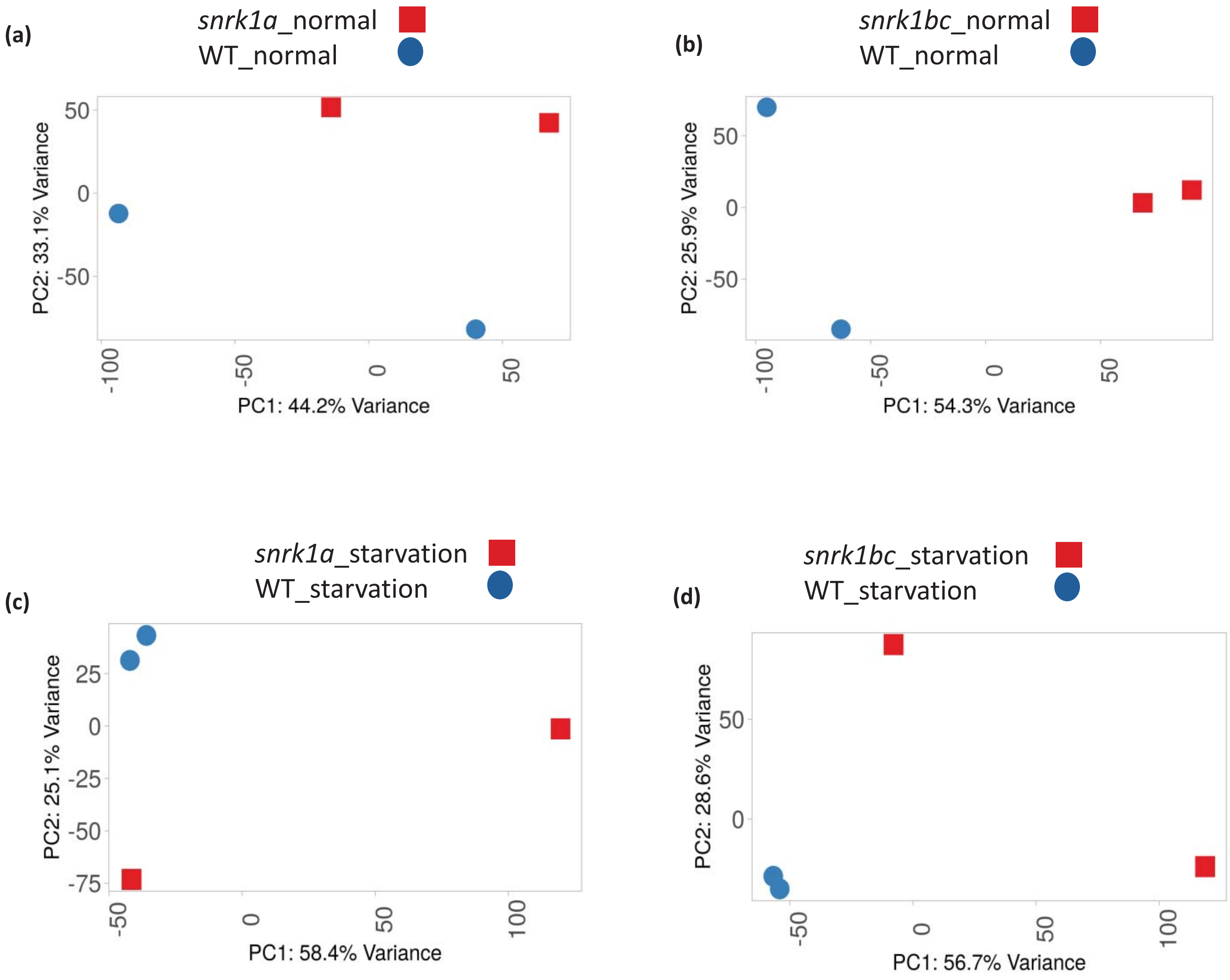

Figure S5. Principal component analysis (PCA) of transcriptomic profiles of *snrk1* mutants and WT under normal or starvation conditions. PCA plots of RNA-seq data (normalized gene count) from 9-d-old WT, *snrk1a*, and *snrk1bc* seedlings grown under normal condition (14-h photoperiod) or subjected to 48 h of continuous darkness after 7 days of normal growth (starvation condition). PCA plots were generated on iDEP web-based tool (Ge et al., 2018).

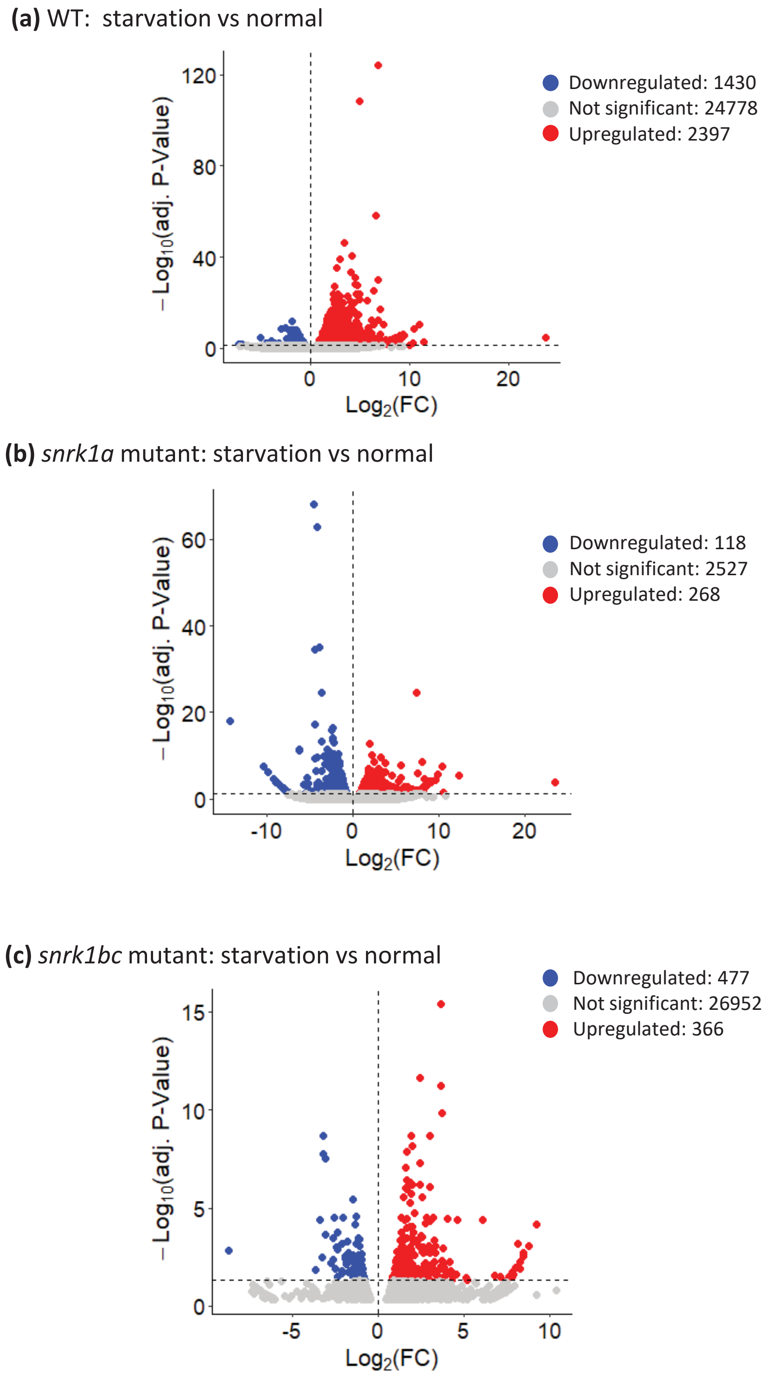

Figure S6. Differential gene expression in WT and *snrk1* mutants under starvation compared to normal growth. Volcano plots showing transcriptomic changes in **(a)** WT, **(b)** *snrk1a*, and **(c)** *snrk1bc* seedlings after 48 h of continuous darkness (starvation) versus those grown in normal condition. Differentially expressed genes (DEGs) were identified based on P-adj.< 0.05 and $|\log_2 FC| > 0$.

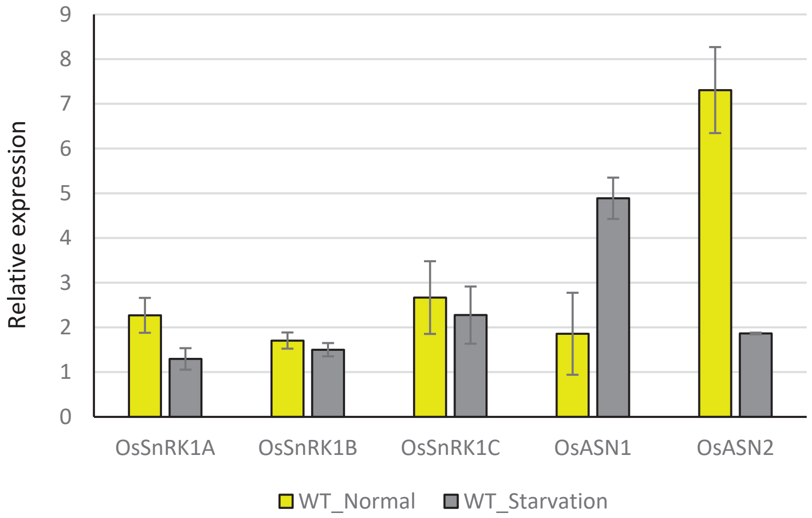

Figure S7: Gene expression analysis of Os*SnRK1α* subunit genes and rice homologs of Arabidopsis dark-induced 6 (*DIN6*). qPCR analysis on the rice *OsSnRK1α* paralogs (A, B, C) and the rice homologs of Arabidopsis *DARK INDUCIBLE 6/ASPARAGINE SYNTHASE 1* (*DIN6/ASN1*) (*OsASN1* and *OsASN2*) in 9-d-old seedlings of WT Kitaake seedlings grown in 14 h photoperiod (normal) or exposed to 2 days of continuous darkness after 7 days of normal growth (starvation). Gene expression was normalized against *OsUBQ2* gene (Os02g0161900). Average of 2 – 3 samples (biological replicates) was plotted with standard deviation shown as error bars.

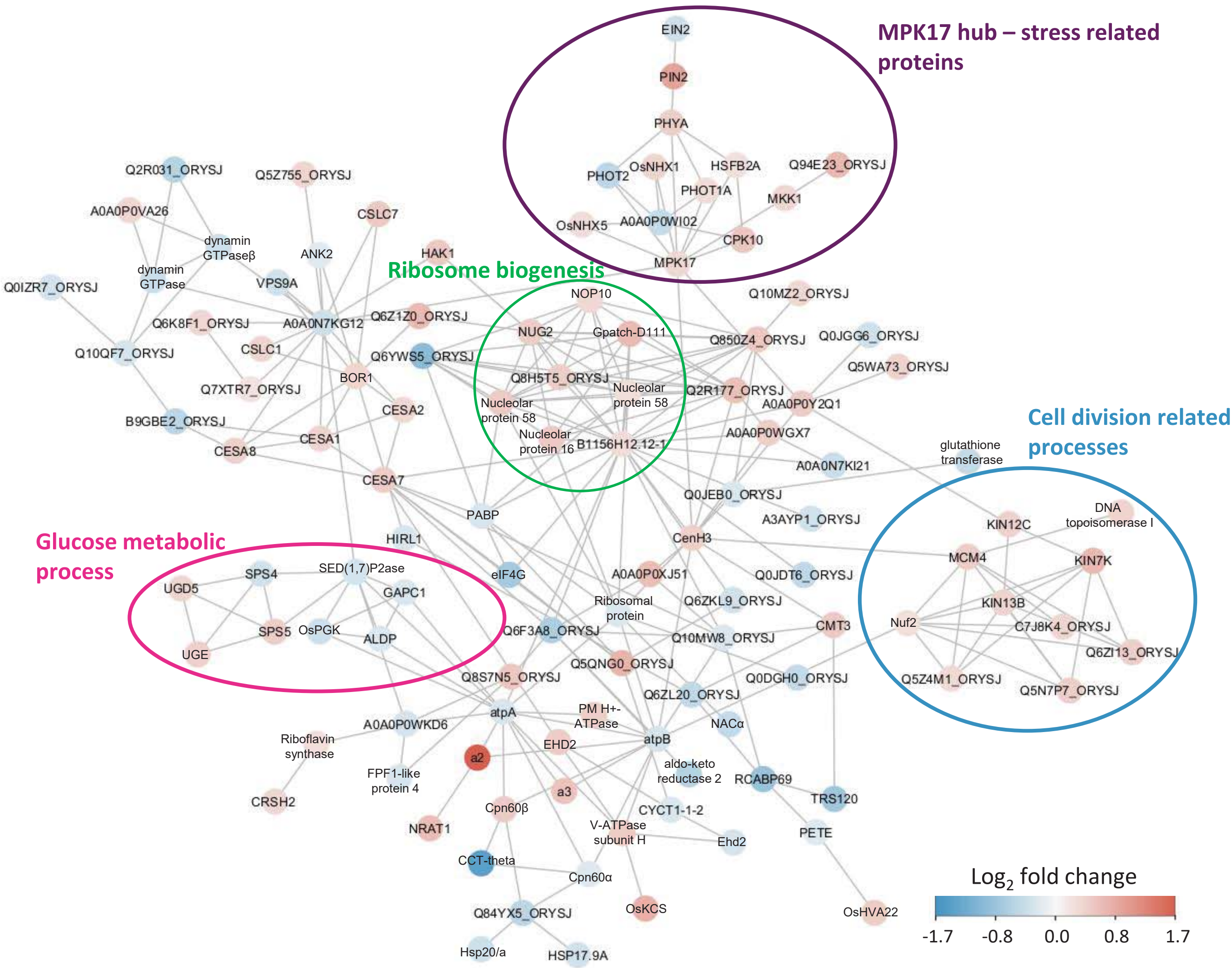

Figure S8: Protein interaction network associated with differentially-abundant phosphosites in *snrk1bc* mutant under extended darkness. STRING-based network analysis of significantly abundant phosphosites in *snrk1bc* seedlings under starvation (p-adj.< 0.055; |$\log_2$ fold change| > 0). Nodes represent proteins and edges represent known or predicted protein–protein interactions. Nodes are color-coded by $\log_2$ fold change: red indicates proteins associated with upregulated phosphosites and blue indicates proteins associated with downregulated phosphosites. Selected networks associated with gene ontology (GO) terms are circled.

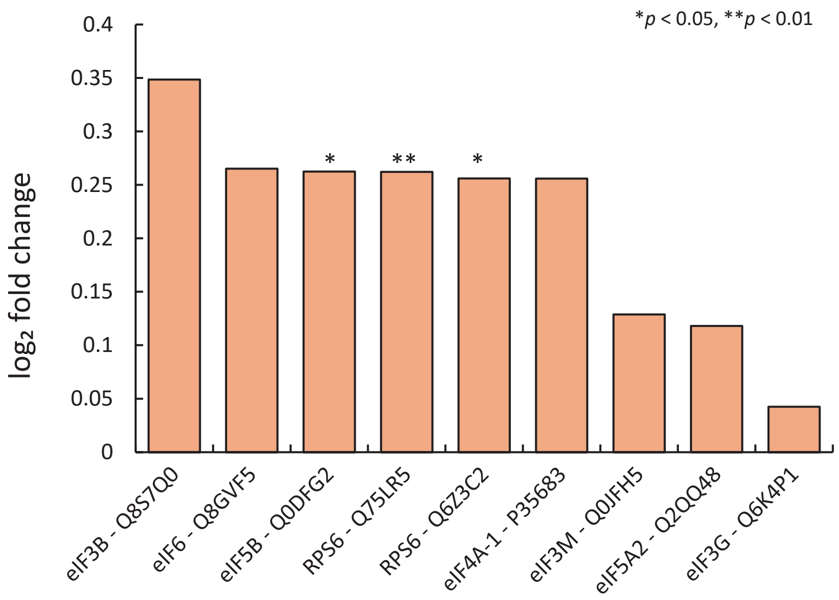

Figure S9: Phosphorylation status of conserved TOR-S6K targets in *snrk1bc*. Enhanced phosphorylation (log_2 fold change) of ribosomal protein S6 (RPS6) and eukaryotic translation initiation factors (eIFs) in *snrk1bc* compared to WT under starvation. Significance (*p*-value) is indicated as * or **. The rest of the phosphosites show non-significant fold-change.

(a)

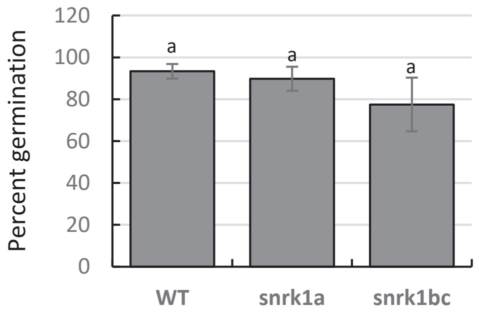

(b)

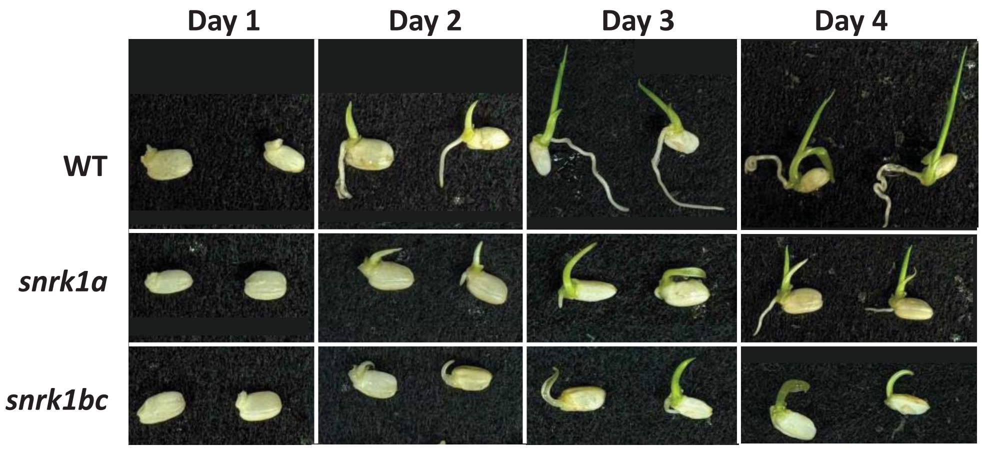

Figure S10: Seed germination on half-strength MS media. (a) percent germination based on number of seeds showing emergence of coleoptile after 3 days on the media at 28°C in 14 h photoperiod (n=70). **(b)** Representative images of germinating seeds of WT and *snrk1* mutants on the media. Note slower rate of shoot and root elongation in *snrk1* mutants, although percent germination is not significantly different (p<0.05) by one-way ANOVA followed by Tukey's HSD test.

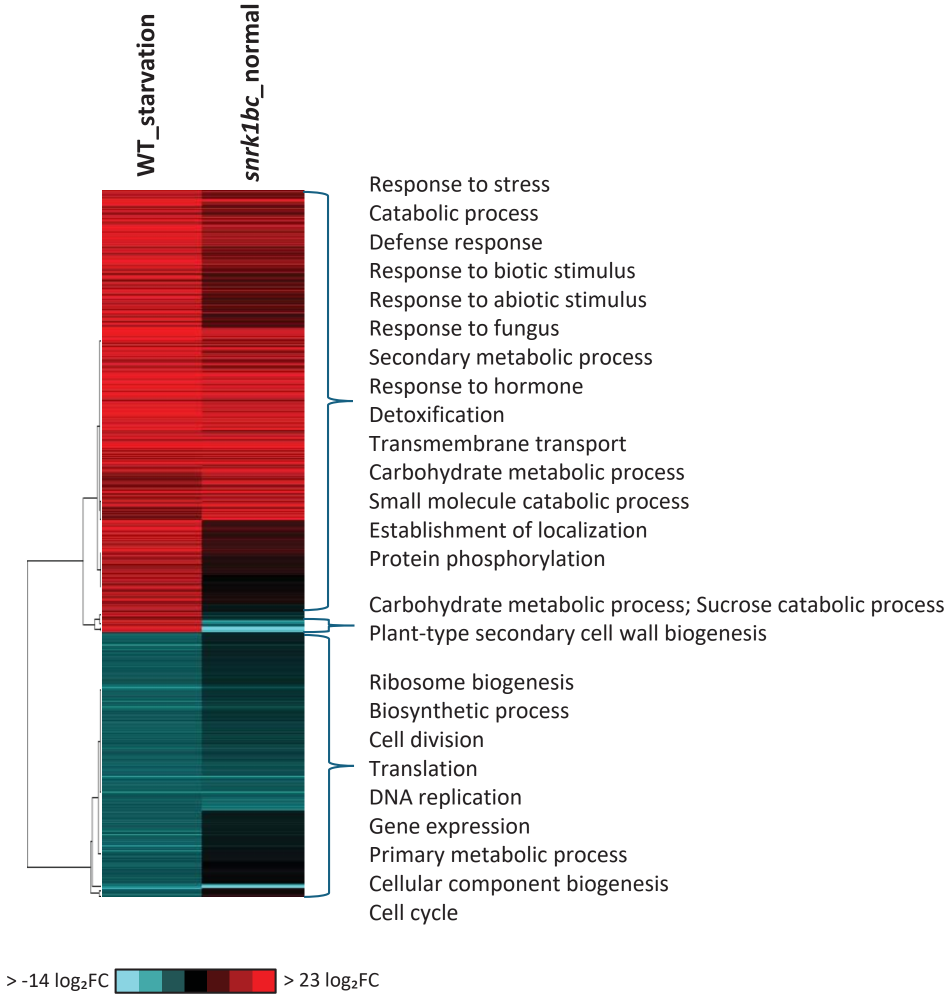

Figure S11: Transcriptomic profile of *snrk1bc* under normal condition mirrors the transcriptomic profile of WT under starvation. Hierarchical clustering of differentially expressed genes in WT under starvation (WT_starvation vs WT_normal) and *snrk1bc* mutant under normal condition (*snrk1bc*_normal vs WT_normal). Genes were clustered based on fold-change, with red indicating upregulation and blue indicating downregulation. Associated gene ontology (GO) terms or functional keywords are annotated.

Proteins	gene name	Sequence window	Phos-AA-Position	*p*-value	p-adj.	log_2FC	-5	-4	-3	-2	-1	0	1	2	3	4	5	Reference
Q6Z411	*RCABP69*	LDQNLAKNNAGEIIG**T**RFETGEVKSTPFQPY	T111	0.000003	0.000702	-0.875404	G	E	I	I	G	**T**	R	F	E	T	G	Hu et al., 2022
Q10NQ3	*VPS9A*	DVREQKSQTLKASRD**S**DVNLSLKDNFQGPGL	S304	0.000092	0.006358	-0.358038	K	A	S	R	D	**S**	D	V	N	L	S	Hu et al., 2022; Hu et al., 2024
Q10NQ3	*VPS9A*	RRDSDASSNPVERVQ**S**ISDLEKKGAAELLKD	S337	0.006362	0.083184	-0.241907	V	E	R	V	Q	**S**	I	S	D	L	E	Hu et al., 2022; Hu et al., 2024
A3AYP1	*Os04g0679400*	AIFQKQVAHAPAELN**S**PRSSAAKPKNPDEIL	S18	0.000535	0.019438	-0.359017	P	A	E	L	N	**S**	P	R	S	S	A	Hu et al., 2022; Hu et al., 2024
Q654U5	*Os06g0247800*	FKGPNTDGGSMRQSN**S**DGALDTMARRPADPE	S722	0.000759	0.022843	-0.400868	M	R	Q	S	N	**S**	D	G	A	L	D	Hu et al., 2022
Q67WN5	*OVP1*	GGAWDNAKKYIEAGA**S**EHARSLGPKGSDCHK	S718	0.001579	0.036164	-0.344055	I	E	A	G	A	**S**	E	H	A	R	S	Hu et al., 2022
Q67WN5	*OVP1*	EGSPGAAAGKDGGAA**S**EYLIEEEEGLNEHNV	S71	0.373253	0.642851	-0.289210	D	G	G	A	A	**S**	E	Y	L	I	E	Hu et al., 2022
Q6ZL20	*OJ1699_E05.17*	LSIAQEAANRSATVQ**S**HEDLARKLKEEMERN	S448	0.001833	0.039365	-0.508572	S	A	T	V	Q	**S**	H	E	D	L	A	Hu et al., 2022
A0A0P0WKD6	*Os05g0291700*	DFSWEKLSTQLAGVA**T**QDSDEVEPKAQIATV	T426	0.003191	0.052913	-0.320021	L	A	G	V	A	**T**	Q	D	S	D	E	Hu et al., 2022

Figure S12: Conserved SnRK1 target motifs identified in rice. Phosphosites corresponding to proteins found in our dataset and previously reported in the literature as SnRK1 targets in rice. Columns include the UniProt protein ID, gene name, the 15-amino-acid phosphosite sequence window (centered on the phosphorylated residue), position of the phospho-acceptor (S or T) within the full-length protein, nominal p-value, adjusted p-value (p-adj.), and $\log_2$ fold change ($\log_2$FC) comparing *snrk1bc* to WT.
