# Supplementary material for: Integrative Transcriptomic and Phosphoproteomic Analysis Reveals Key Components of the SnRK1 Signaling Network in Rice
Source: Plant Direct. 2025 Nov 17;9(11):e70120. doi: 10.1002/pld3.70120 (PMC12623061; doi:10.1002/pld3.70120)
Supplement: Supplementary file 1 — Data S1: Peer Review. [file PLD3-9-e70120-s004.pdf]

Plant Direct
Open Access

Integrative transcriptomic and phosphoproteomic analysis reveals key components of SnRK1 signaling network in rice

Vibha Srivastava, Ms. Maria C. Faria-Bates , Mr. Chandan Maurya , Dr. Muhammed Jamsheer K

Decision Letter Round 1:

September 24, 2025

Dr. Vibha Srivastava

University of Arkansas Division of Agriculture

115 Plant Science bldg., University of Arkansas

Fayetteville, Arkansas 72701

RE: Integrative transcriptomic and phosphoproteomic analysis reveals key components of SnRK1 signaling network in rice

Dear Dr. Srivastava:

Thank you for submitting to Plant Direct. All required reviews have been returned and we have now finished our evaluation of your manuscript. In light of the reviewers' and editor's comments, further revisions are needed before the paper can be accepted for publication in Plant Direct.

Please view the editors' and reviewers' comments below and use their suggestions as a guide while you work on your revision.

When uploading the revised version of this article, please be sure to include the following:

-A word document that contains your response to the reviewers. You should respond to each reviewer comment and note the changes made to the manuscript. If you do not agree with a reviewer's comment and choose not to make a suggested revision, please explain why. Please try to provide as complete an answer as possible to each reviewer's criticisms.

-A tracked changes document with each change highlighted

- A clean version of the latest version of the manuscript

To upload your revision, please click the link below.

https://plantdirect.msubmit.net/cgi-bin/main.plex?el=A5Lr5pW2A4DRN2I6A9ftdFYw5KXPgzqDEfSgnZMHpSAZ

In order to provide as timely a service as possible, we ask that your revision is resubmitted within three months after receipt of this request. If an extension is needed, please send a request, along with a brief explanation, to the editorial office at plantdirect@wiley.com .

Please note that, in addition to publishing reviewer comments, the author's responses to review comments will also be published alongside the final version of the paper. If you would not like the author's responses to be published, please contact the editorial office at plantdirect@wiley.com .

Thank you very much for giving us an opportunity to review your work. I look forward to receiving the next version.

Sincerely,

Yan Lu

Esther van der Knaap

Editor, Plant Direct

-- Editor comments:

-- Reviewer comments:

Reviewer #1:

This relevant study describes the effects of SnRK1 loss of function in rice, confirming key roles for this kinase in plant growth in favourable conditions, in the adaptation to carbon and energy starvation, and in defence (against rice blast disease). After general growth phenotyping of vegetative (seedling) and reproductive development and rice blast response of previously generated and described snrk1a and snrk1bc SnRK1□ CRISPR/Cas9 KO mutants (Pathak et al., 2022), transcriptomic, proteomic and phosphoproteomic analyses of these mutants in control and (for the transcriptomics) starvation conditions provide insight in known/conserved and putative novel (direct and indirect) transcriptional and posttranslational targets and downstream protein networks. Previously, the focus has been primarily on SnRK1A. This study now highlights an important role for the other catalytic subunits in rice seedling's starvation response. While the analyses are sometimes confirmatory and/or descriptive (without confirmation or mechanistically linking targets to phenotypes), this study is an interesting and very valuable resource for the research community for future studies on an important kinase in an important crop. It is generally well written and presented.

More specific (minor) comments:

. Introduction: Maybe additional studies on SnRK1 function in rice/cereals should be introduced here for completeness (e.g. on the role of SnRK1A in sugar signaling during germination and seedling growth or the studies by Hu et al., discussed later).

. p5-6: The reduced biomass of snrk1bc seedlings, especially in starvation conditions where no effect on shoot or root length is observed, is intriguing. Can the authors discuss this somewhat more and maybe provide a possible explanation?

. p6, line 4: The authors (justly) suggest sub-functionalization of the different catalytic subunits based on the phenotypes of the two mutants. Can differential expression patterns of the three catalytic subunits also be considered? Are they also similar for SnRK1B and SnRK1C. Was there no seed (endosperm)-specific expression reported for SnRK1B?

. p6, line 14-18: Somewhat confusing to discuss the YT16 (YT1 in the figure) susceptible cultivar without introduction. I assume it is included as control.

. p6, line 27: A bit strange to introduce the additional mutant lines after a summary of the results. What is meant by 'representing the second allele'? How are they different? Labels in Fig. S4 (e) need correction. It looks like mutant snrk1a.2 barely produced seeds?

. p7, RNAseq analysis: comprehensive analysis with interesting and surprising (counter-intuitive) effects of SnRK1 mutation on gene expression in control conditions, confirming a key role for this kinase in normal growth and development in non-stress conditions. Materials and Methods mention that two biological replicates were used each consisting of only 2-5 seedlings. Is that correct?

. p9, phosphoproteomics: control conditions were not included. Why? Would this not have been helpful to study the (SnRK1-dependent and -independent) starvation-specific effects? Interesting analysis and discussion of putative direct and indirect targets. A study on VPS9a's role in the GS/GOGAT cycle and autophagy under nutrient starvation was recently published in Plant J. (Yu et al., 2025). Maybe relevant and interesting to discuss here?

Reviewer #2:

This study by Faria-Bates and colleagues used CRISPR/Cas9-generated rice snrk1 mutants and integrated transcriptomic, proteomic, and phosphoproteomic approaches to elucidate the role of SnRK1 in growth regulation and stress responses under both normal and starvation conditions. Overall the project is very well-executed and results are clearly presented and summarised. Some of the minor suggestions and comments from my side are highlighted below.

Abstract, Introduction and Discussion sections are nicely written. Authors have provided sufficient background information and discussed the results with calrity. moreover, references are up-to-dated and relevant.

On P19 L15 in M&M section, I would suggest adding reference of the genome assembly you used to map the RNA-seq data.

P19 L18-24: What was the concentration of Chla and Chlb contents? How did you calculate them? Calculate them and add their methods and results.

In the results sections on P3 L5, I would suggest performing and adding results of histochemical analysis to support defence-related results and the role of SnRK1.

Please submit all the sequencing data to any data repository, e.g., NCBI, and provide its submission/reference ID.

Plant Direct
Open Access

Decision Letter Round 2:

October 24, 2025

Dr. Vibha Srivastava

University of Arkansas Division of Agriculture

115 Plant Science bldg., University of Arkansas

Fayetteville, Arkansas 72701

MSID: 2025-01831-TWR1

MS TITLE: Integrative transcriptomic and phosphoproteomic analysis reveals key components of SnRK1 signaling network in rice

Dear Dr. Vibha Srivastava:

I am pleased to inform you that your manuscript "Integrative transcriptomic and phosphoproteomic analysis reveals key components of SnRK1 signaling network in rice" has been accepted for publication in Plant Direct.

Your article will appear online in the next available issue of Plant Direct. To ensure your article gets published as quickly as possible, please pay attention to the steps detailed below. We have found that most of the delays happen at this stage, especially at the payment stage, so please respond as quickly as possible when prompted.

License Agreement: Once your article has been accepted it will move to Production and undergo admin and file checking - you may receive an email with any queries we have at this stage. When all required items are received by the publisher and queries resolved, the corresponding author will receive an email from Wiley's Author Services system which will ask them to log on at https://authorservices.wiley.com/bauthor and will present them with the appropriate license for completion. Your article cannot be published until both the signed license agreement and payment of the article fee have been received.

Payment of the Open Access Article Publication Fee: All articles published in Plant Direct are fully open access: immediately and freely available to read, download and share. Plant Direct charges a publication fee to cover the publication costs. The corresponding author for this manuscript should have already received a quote from the payments team (cs-openaccess@wiley.com) with the estimated article publication fee; please email cs-openaccess@wiley.com if this has not been received. The corresponding author should log on to the Wiley Author Services site, where the publication fee can be paid by credit card or an invoice. Pro Forma can also be requested. Payment of the publication charge must be received before the article will be published online.

Proofs: You will have the opportunity to look over your paper once more when you receive the author proofs for your article. The proofs will be with you in approximately two weeks. Please note that, in addition to publishing reviewer comments, the author's responses to review comments will

also be published alongside the final version of the paper. If you would not like the author's responses to be published, please contact the editorial office at plantdirect@wiley.com .

Promotion of your article: You can help your research get the attention it deserves! Check out Wiley's free Promotion Guide for best-practice recommendations for promoting your work at www.wileyauthors.com/eeo/guide. And learn more about Wiley Editing Services which offers professional video, design, and writing services to create shareable video abstracts, infographics, conference posters, lay summaries, and research news stories for your research at www.wileyauthors.com/eeo/promotion.

Thank you again for your contribution to Plant Direct. If you have any questions, feel free to contact the editorial office at plantdirect@wiley.com .

Sincerely,

Yan Lu

Esther van der Knaap

Editor, Plant Direct

Response Letter Round 1:

Response to the reviewers' comments:

Dear Editors,

Thank you for sending comprehensive reviews on our manuscript. We have considered all comments and revised the manuscript. Below, please find our response to each of the comment. In addition, we found mistakes in gene IDs, which we corrected in this revised manuscript (page 5, line 19-20.

Best regards,

Vibha Srivastava

Corresponding author

COMMENTS FOR THE AUTHOR:

Reviewer #1: This relevant study describes the effects of SnRK1 loss of function in rice, confirming key roles for this kinase in plant growth in favourable conditions, in the adaptation to carbon and energy starvation, and in defence (against rice blast disease). After general growth phenotyping of vegetative (seedling) and reproductive development and rice blast response of previously generated and described snrk1a and snrk1bc SnRK1[illegible] CRISPR/Cas9 KO mutants

(Pathak et al., 2022), transcriptomic, proteomic and phosphoproteomic analyses of these mutants in control and (for the transcriptomics) starvation conditions provide insight in known/conserved and putative novel (direct and indirect) transcriptional and posttranslational targets and downstream protein networks. Previously, the focus has been primarily on SnRK1A. This study now highlights an important role for the other catalytic subunits in rice seedling's starvation response. While the analyses are sometimes confirmatory and/or descriptive (without confirmation or mechanistically linking targets to phenotypes), this study is an interesting and very valuable resource for the research community for future studies on an important kinase in an important crop. It is generally well written and presented.

Reviewer’s Comment 1. Introduction: Maybe additional studies on SnRK1 function in rice/cereals should be introduced here for completeness (e.g. on the role of SnRK1A in sugar signaling during germination and seedling growth or the studies by Hu et al., discussed later).

Response: Thank you for this suggestion! We have summarized studies related to the role of SnRK1 in rice, wheat, maize developmental processes and added three new references (Bledsoe et al 2017; Kumar et al., 2024; Zhang et al., 2001). See page 4, lines 14 - 29.

Reviewer’s Comment 2. p5-6: The reduced biomass of snrk1bc seedlings, especially in starvation conditions where no effect on shoot or root length is observed, is intriguing. Can the authors discuss this somewhat more and maybe provide a possible explanation?

Response: snrk1bc seedlings under starvation developed very thin seedlings consisting of visibly thin leaves and culm, indicating reduced leaf width and tissue thickness. This likely contributes to lower biomass despite no effect on seedling length. We have moved this paragraph in this section to clarify biomass defect in snrk1bc under starvation. (Page 6, line 10-11).

Reviewer’s Comment 3. p6, line 4:

The authors (justly) suggest subfunctionalization of the different catalytic subunits based on the phenotypes of the two mutants. Can differential expression patterns of the three catalytic subunits also be

considered? Are they also similar for SnRK1B and SnRK1C. Was there no seed (endosperm)-specific expression reported for SnRK1B?

Response: Reviewer makes an excellent point and rightly suggests that OsSnRK1A, B/C genes fit the definition of subfunctionalization as deletion of OsSnRK1A or OsSnRK1BC leads to fitness cost (Birchler 2025).

Can differential expression patterns of the three catalytic subunits also be considered?

Yes, these genes show differential expression (Response Fig. 1); however, whether that is entirely the basis of functional specialization, cannot be concluded mainly because all three genes are mostly expressed in all tissues despite at differential levels.

Are they also similar for SnRK1B and SnRK1C?

Concurring with high sequence homology between OsSnRK1B and C, their expression patterns are more similar to each other than with OsSnRK1A. In fact, there doesn't seem to be too many tissue types where

OsSnRK1C shows higher expression

than OsSnRK1B (Response Fig. 1).

Response Fig. 1: Expression patterns of OsSnRK1A, B, C

in different tissues based on publicly available RNAseq

(https://plantrnadb.com/ricerna/). Number in parentheses

represent number of RNAseq datasets for each tissue.

Datasets differ in the developmental stage and treatment of

tissues as well as the genotypes.

3

Was there no seed (endosperm)-specific expression reported for SnRK1B?

OsSnRK1B and C both show high expression in seeds and endosperm (Response Fig. 1). This is also stated in the manuscript page 15, line 24-26.

It is not clear to us why the reviewer raised this question. Our phenotypic analysis shows low number of seeds per panicle in both snrk1 mutants but no significant difference in 100 seed weight. These observations suggest that both mutants have low fertility, but when seeds form, they are more or less normal. Therefore, seed filling under greenhouse condition is not significantly affected by snrk1 mutation. However, whether this function would be affected under stress, cannot be determined in our study.

Reference:

Birchler JA. When is it subfunctionalization and when is it not? G3 (Bethesda). 2025 Jan 8;15(1):jkae269.

Reviewer's Comment 4. p6, line 14-18: Somewhat confusing to discuss the YT16 (YT1 in the figure) susceptible cultivar without introduction. I assume it is included as control.

Response: We have added a sentence about disease susceptibility in YT16 (page 6, line 28-29). It carries a mutant pi-ta allele and shows high susceptibility to M. oryzea. We checked Fig. 1 and Fig. S4 and their figure legends, in both figures YT16 is correctly labeled.

Reviewer's Comment 5. p6, line 27: A bit strange to introduce the additional mutant lines after a summary of the results. What is meant by 'representing the second allele'? How are they different? Labels in Fig. S4 (e) need correction. It looks like mutant snrk1a.2 barely produced

seeds?

Response: We want to keep the manuscript within acceptable word limit consisting of appropriate number of figures. If we added “second allele” in the main results, the section would have been much longer and sounded repetitive. However, to link the phenotype with the gene, it is important to analyze at least two independent mutant lines. For this reason, we added the second mutant line for each of OsSnRK1A and OsSnRK1BC referred to as snrk1a.2 and snrk1bc.2 in the supplementary file and only briefly discussed it in the main text. As stated on page 7, line 8-10 second allele is derived from a new mutant line. To clarify, we have added snrk1a.2 and snrk1bc.2 mutant sequences in Supplementary File S2. We were unable to find a mistake in Fig. S4(e) and yes, snrk1a.2 barely produced seeds just as snrk1a mutant did (1.55 seeds/panicle in snrk1a, 2.6 seeds/panicle in snrk1a.2, compared to 28.2 seeds/panicle in WT).

Reviewer’s Comment 6. p7, RNAseq analysis: comprehensive analysis with interesting and surprising (counter-intuitive) effects of SnRK1 mutation on gene expression in control conditions, confirming a key role for this kinase in normal growth and development in non-stress conditions. Materials and Methods mention that two biological replicates were used each consisting of only 2-5 seedlings. Is that correct?

4

Response: Yes, two separate bulks consisting of variable number of seedlings were used. This is mainly because we tested each seedling for the presence of homozygous mutations by Sanger sequencing and only the sequence-verified seedlings were selected.

Reviewer’s Comment 7. p9, phosphoproteomics: control conditions were not included. Why? Would this not have been helpful to study the (SnRK1-dependent and -independent) starvationspecific effects? Interesting analysis and discussion of putative direct and indirect targets. A study on VPS9a's role in the GS/GOGAT cycle and autophagy under nutrient starvation was recently published in Plant J. (Yu et al., 2025). Maybe relevant and interesting to discuss here?

Response: Phosphoproteomic analysis was carried out only under starvation conditions, because this is when SnRK1 signaling is strongly activated. We agree with the reviewer that phosphoproteomics in the control conditions would also be interesting, but since the phenotypic defects in the snrk1bc seedlings under control conditions were not as severe as under starvation

(Fig. 1), we decided to focus on starvation condition.

Thank you for bringing Yu et al. study to our attention, we have added a paragraph showing the relevance of VPS9a and SnRK1 with nutrient stress response (page 12, line 19-22)

____

Reviewer #2: This study by Faria-Bates and colleagues used CRISPR/Cas9-generated rice snrk1 mutants and integrated transcriptomic, proteomic, and phosphoproteomic approaches to elucidate the role of SnRK1 in growth regulation and stress responses under both normal and starvation conditions. Overall the project is very well-executed and results are clearly presented and summarised. Some of the minor suggestions and comments from my side are highlighted below.

Abstract, Introduction and Discussion sections are nicely written. Authors have provided sufficient background information and discussed the results with calrity. moreover, references are up-to-dated and relevant.

Reviewer’s Comment 1. On P19 L15 in M&M section, I would suggest adding reference of the genome assembly you used to map the RNA-seq data.

Response: Thanks for pointing out this discrepancy. We have added this information on page 20, line 1-2.

Reviewer’s Comment 2. P19 L18-24: What was the concentration of Chla and Chlb contents? How did you calculate them? Calculate them and add their methods and results.

Response: We calculated total chlorophyll by summing up Chla and Chlb contents using the formula given in the methods. To clarify this, we have modified the text on page 20, line 10-11.

Reviewer’s Comment 3. In the results sections on P3 L5, I would suggest performing and adding results of histochemical analysis to support defence-related results and the role of SnRK1.

Response: The goal of this study was to test whether snrk1 mutation leads to a change in disease response. For this, we chose rice blast disease, the most devastating rice disease caused by rice blast fungus Magnaporthe oryzea. We conducted this study in consultation with rice pathologists using well-established methods of detached leaf spotting and detached leaf spray. It is our understanding that histochemical analysis is not needed for determining disease severity. Filipe et al (2018) also used rice blast disease assay on ossnrk1 mutants by simply testing the severity

of disease. Cao et al. (2024) carried out microscopic analysis to calculate types of invasive hyphae on WT vs ossnrk1 mutants (categorized as type 1 – 4). They found that all types of invasive hyphae are seen on ossnrk1a mutant. Therefore, we feel that histological or histochemical analysis will not provide critical information on disease severity.
However, we carried out microscopic analysis to monitor spore germination and formation of invasive hyphae from the appressoria using sheath inoculation assay (Response Fig. 2). This analysis provided histological view of disease response and showed differences in necrosis and invasive hyphae and appressoria between genotypes. These images were captured by smart phone. Specifically, on WT_Kitaake, invasive hyphae are not visible. On YT16, on the other hand, multiple invasive hyphae are seen (blue arrows). On snrk1a and snrk1bc mutants, browning is notable indicating necrosis and, in this image of snrk1a, hyphae from appressoria are also visible. Please note that invasive hyphae were also seen on snrk1bc but not captured in this image.
Response Fig. 2: Microscopic observation of Magnaporthe oryzae Guy11 infection in rice leaf sheaths of WT, snrk1a, snrk1bc, and YT16 (susceptible control). Sheath inoculation assays were performed to visualize the formation of appressoria and invasive hyphae as well as necrosis. Samples were collected 48–72 h after inoculation and examined under a light microscope. Images were captured by smart phone
Reviewer’s Comment 4. Please submit all the sequencing data to any data repository, e.g., NCBI, and provide its submission/reference ID.
6
Response: We have initiated the data submission process into the NCBI Sequence Read Archive (SRA) under the submission ID SUB15675617 on September 29, 2025. However, due to the U.S. government shutdown that began on October 1, 2025, the NCBI submission portal is temporarily closed, and we were unable to complete the upload of the sequencing files. We also initiated the process to obtain a GEO accession number in early October 2025, but since the government shutdown was already in effect, the GEO system was not issuing accession numbers. As soon as the NCBI system resumes normal operation, we will finalize the data upload and provide the accession number in the revised manuscript.
